# Hydrophilic Shell Matrix Proteins of *Nautilus pompilius* and the Identification of a Core Set of Conchiferan Domains

**DOI:** 10.3390/genes12121925

**Published:** 2021-11-29

**Authors:** Davin H. E. Setiamarga, Kazuki Hirota, Masa-aki Yoshida, Yusuke Takeda, Keiji Kito, Makiko Ishikawa, Keisuke Shimizu, Yukinobu Isowa, Kazuho Ikeo, Takenori Sasaki, Kazuyoshi Endo

**Affiliations:** 1 Department of Applied Chemistry and Biochemistry, National Institute of Technology (KOSEN), Wakayama College, Gobo 644-0023, Japan; hirota-kazuki96@um.u-tokyo.ac.jp; 2 Graduate School of Sciences, The University of Tokyo, Bunkyo-ku, Tokyo 113-0033, Japan; makiko@eps.s.u-tokyo.ac.jp (M.I.); k.shimizu.bio14@gmail.com (K.S.); yukinobu.isowa@gmail.com (Y.I.); endo@eps.s.u-tokyo.ac.jp (K.E.); 3 The University Museum, The University of Tokyo, Tokyo 113-0033, Japan; ytakeda@sci.hokudai.ac.jp (Y.T.); sasaki@um.u-tokyo.ac.jp (T.S.); 4 Marine Biological Science Section, Education and Research Center for Biological Resources, Faculty of Life and Environmental Science, Shimane University, Unnan 685-0024, Japan; mayoshida@life.shimane-u.ac.jp; 5 Graduate School of Science, Hokkaido University, Sapporo 060-0810, Japan; 6 Department of Life Sciences, School of Agriculture, Meiji University, Kawasaki 214-8571, Japan; kito@isc.meiji.ac.jp; 7 Faculty of Animal Health Technology, Yamazaki University of Animal Health Technology, Hachiouji 192-0364, Japan; 8 Graduate School of Agriculture and Life Sciences, The University of Tokyo, Yayoi, Tokyo 113-8657, Japan; 9 Shimoda Marine Research Center, University of Tsukuba, Shimoda 415-0025, Japan; 10 Center for Information Biology, National Institute of Genetics, Mishima 411-8540, Japan; kikeo@nig.ac.jp

**Keywords:** biomineralization, multiomics, proteomics, shell evolution, Mollusca, Cephalopoda

## Abstract

Despite being a member of the shelled mollusks (Conchiferans), most members of extant cephalopods have lost their external biomineralized shells, except for the basally diverging Nautilids. Here, we report the result of our study to identify major Shell Matrix Proteins and their domains in the Nautilid *Nautilus pompilius,* in order to gain a general insight into the evolution of Conchiferan Shell Matrix Proteins. In order to do so, we performed a multiomics study on the shell of *N. pompilius*, by conducting transcriptomics of its mantle tissue and proteomics of its shell matrix. Analyses of obtained data identified 61 distinct shell-specific sequences. Of the successfully annotated 27 sequences, protein domains were predicted in 19. Comparative analysis of *Nautilus* sequences with four Conchiferans for which Shell Matrix Protein data were available (the pacific oyster, the pearl oyster, the limpet and the *Euhadra* snail) revealed that three proteins and six protein domains were conserved in all Conchiferans. Interestingly, when the terrestrial *Euhadra* snail was excluded, another five proteins and six protein domains were found to be shared among the four marine Conchiferans. Phylogenetic analyses indicated that most of these proteins and domains were probably present in the ancestral Conchiferan, but employed in shell formation later and independently in most clades. Even though further studies utilizing deeper sequencing techniques to obtain genome and full-length sequences, and functional analyses, must be carried out in the future, our results here provide important pieces of information for the elucidation of the evolution of Conchiferan shells at the molecular level.

## 1. Introduction

Many metazoans have evolved various biomineralized tissues, both internally and externally [1]. Despite its maintenance cost, many metazoan species have opted to retain the presence of such tissues because they are deemed useful, for example, for structural and morphological support, mineral ions storage and protection and defense from predators and environmental factors [2,3]. Among extant metazoans, two phyla have anciently evolved and are still retaining their external biomineralized shells: the mollusks (Mollusca) and the brachiopods (Brachiopoda) [1]. Most members of these calcifying organisms live in marine environment, where calcium and carbonate ions are easily available as sources of the mineralized tissues [4].

With ca. 85,000 extant members, the phylum Mollusca is one of the most successful metazoan groups. Recent phylogenomics studies have shown that a monophyletic Mollusca is comprised of two groups, Aculifera (polyplacophorans and aplacophorans) and the biomineralized, external shell-forming Conchifera (=“the Conchiferans”). The latter group is comprised of five families grouped further into two monophyletic clades: the monoplacophorans + cephalopods clade and the scaphopods + gastropods + bivalves clade [5,6,7,8]. Conchiferans’ ability to form mineralized external shells was acquired very early in their evolution in the Cambrian [9,10]. The Conchiferan shell is arguably the most well-studied biomineralized external structure [11]. Mineralogy and microstructure studies have revealed that Conchiferan shells are mainly based on calcium carbonate and composed of multiple calcified layers (such as the prismatic and nacreous layers) and one organic layer (the periostracum). The mechanism of shell formation, which includes several distinct steps such as secretion of various proteins related to mineral depositions by the mantle tissue, crystal formation breakage, pigmentation, etc., is also shared among the Conchiferans [11]. Meanwhile, recent development in genomics, transcriptomics, proteomics and other “-omics” approaches have allowed for detailed molecular characterizations of shell formation and biomineralization. For example, multiomics approaches, such as integrating transcriptomics or Expressed Sequence Tag (EST) analysis with proteomics, have revealed a putative list of genes involved in biomineralization processes in mollusks [12,13,14,15,16]. Many of such proteins are present in trace amounts inside the shell, and thus called the Shell Matrix Proteins (SMPs). Despite their small amount, the SMPs apparently have essential roles in shell formation and structural maintenance, such as calcium carbonate nucleation, crystal growth and choice of calcium carbonate polymorphs [17,18].

**Figure 1:**
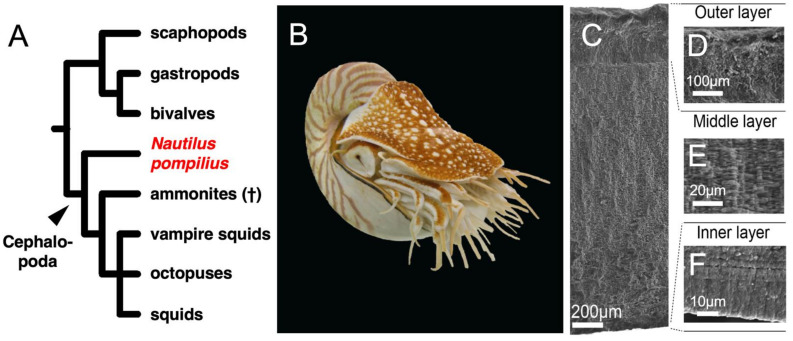
(**A**) Phylogeny of Conchiferans including *Nautilus pompilius*. (**B**) *N. pompilius.* (**C**) The microstructures of the shell of *N. pompilius*. In detail: (**D**) Outer prismatic layer, (**E**) Middle prismatic layer and (**F**) Inner prismatic layer.

Among the five Conchiferan orders, the evolution of the cephalopod shell is arguably the most intriguing. While the group includes famous extinct members with univalve shells such as the ammonites and belemnites, almost all extant cephalopods internalized, reduced or completely lost their shells (such as seen in some cuttlefishes, squids and octopods). Only *Nautilus* (Figure 1A,B), the last surviving genus of the basally diverging Nautilids (Nautilida: Nautilidae) (±416 MYA, i.e., Silurian/Devonian boundary) still have its external calcified shells [19]. The *Nautilus* shell also shows similar microstructures to those of other Conchiferan shells ([20], Figure 1C). For example, the outer shell wall of *Nautilus pompilius* is also composed of three layers of minerals, the outer and inner prismatic layers, and the nacreous layer in between (Figure 1C–F; [11,21]). Meanwhile, another member of the cephalopods, the argonauts (Octopodiformes: Argonautidae) also have an external calcified shell. However, this shell is considered as not a “true” shell because it lacks the microstructures of one, brittle and most likely acquired secondarily from a shell-less Octopodiform ancestor during the evolution of this group [22,23,24]. 

Many studies on shell biomineralization genes, proteins and protein domains have been carried out on bivalves and gastropods. However, in order to obtain a more general insights on the origin and evolution of the SMPs in the Conchiferans, information from the Cephalopods is crucial. Therefore, in this study, we conducted a multiomics study on the hydrophylic proteins extracted from the shell matrix of the Nautilid *Nautilus pompilius* (Figure 1B). The results allowed us to identify putative conserved sets of proteins and protein domains in the Conchiferan SMPs, which then allowed us to further discuss the finding’s implication on the understanding of the evolution of Conchiferan SMPs and shell formation.

## 2. Materials and Methods

### 2.1. Sample Collections, Total RNA Extraction from Mantle Tissues and Total Shell Protein Extraction

Three individuals of *Nautilus pompilius* were obtained from a local aquarium shop dealer in Japan. The samples were obtained from The Philippines (Tokyo, Japan). We obtained these samples at the end of 2011 and beginning of 2012, before the inclusion of this species in the CITES list and thus prior to the protected status of this species under the Washington agreement. First, we sedated the individuals in 2% ethanol in cold sea water for ca. 10 min [25]. Afterward, we removed the shells from the individuals, and dissected out pieces of the mantle tissue (ca. 25–35 mg each) on ice, and stored them in ISOGEN (Nippon Gene Co. Ltd., Tokyo, Japan) at −80 °C. The mantle tissues were extracted from the dorsal part and ventral part of the outermost rim of the mantle, positioned behind the hood, at the part directly touching the rim of the shell. Total RNA was extracted from the tissue samples using ISOGEN and the RNeasy kit (Qiagen, Tokyo, Japan) and was stored in −80 °C until further transcriptome analyses. The rest of the body of the individuals were euthanized by freezing them in −80 °C, and then preserved in formalin, to be later stored as vouchered specimens at The University Museum, The University of Tokyo, Japan. 

The extraction process of the total shell protein was as follows. The shell of one of the individuals was first shattered into pieces using a hammer. The shell pieces were cleaned from any organic tissue by incubation in a 2M NaOH overnight, and a thorough washing with Milli-Q water 10 times. Cleaned shell pieces were then ground into powder, and then slowly decalcified using 0.5 M EDTA as the chelating agent, at 4 °C for 3 days. Total hydrophilic proteins of the shell were extracted using the 3 kDa Amicon Ultra Centrifugal Filter Unit. Extracted protein were then stored at −80 °C until further analyses.

### 2.2. Multiomics Analyses of the Shell Matrix Proteins of Nautilus pompilius

Transcriptome sequencing of the mRNA extracted from the seven tissue samples, using the Ion Torrent PGM next generation sequencing platform (Thermo Fisher Scientific, Waltham, MA, USA) was outsourced to the Center for Omics and Bioinformatics, The University of Tokyo. Obtained raw reads from the seven tissue samples were combined and assembled altogether using the CLC assembly cell with the default settings on Maser Computing System (Data Center for Cell Innovation, National Institute of Genetics) [26]. The Maser analytical pipelines (http://cell-innovation. nig.ac.jp/; accessed on 1 November 2013) were then used for functional estimations of the assembled CLC contigs. For expression profiling, FASTQ reads were aligned to the CLC contigs using the TMAP mapping program (https://github.com/iontorrent/TS/tree/master/Analysis/TMAP; accessed on 1 November 2013).

After digestion into short peptides by trypsin (Promega, Tokyo, Japan), extracted total shell protein samples were analyzed using a Liquid Chromatography–Mass spectrometry/Mass spectrometry (LC-MS/MS) system (DiNa nanoLC system) (KYA Technologies, Tokyo, Japan) and an LTQ Orbitrap Mass Spectrometer (Thermo Fisher Scientific)). Identification of obtained spectra was conducted by doing a search on a self-prepared protein sequence database using the spectra as queries, on the SEQUEST program in Proteome Discoverer version 1.2 (Thermo Fisher Scientific). The self-made protein sequence database was built as follows: First, assembled transcriptome contig data from the mantle tissue were translated and then fragmented into hypothetical peptides in silico to simulate digestion by trypsin. Afterward, hypothetical molecular masses and spectra of the hypothetical peptides were calculated. The hypothetical spectrum data were then matched to the actual experimental spectrum data of the Shell Matrix Protein (SMP) polypeptides obtained from the LC-MS/MS analyses, resulting in the identification of candidate protein sequences. Only transcriptome-based protein sequences (the hypothetical protein sequences) matched by at least two LC MS/MS polypeptides were selected as potential SMPs. Detailed methods and parameters for the analyses were described in [4,27,28].

### 2.3. Characterizations of the Shell Matrix Proteins of Nautilus pompilius

Sequence annotation was performed by conducting BLASTp and BLASTx searches on the nr databases of Genbank and a database of published Conchiferan Shell Matrix Protein sequences, which we compiled ourselves by expanding the dataset of Arivalagan et al. (2017) and Feng et al. (2017) (Supplementary Table S1) [29,30]. Domain searches were performed using multiple online tools: SMART (http://smart.embl-heidelberg.de/; accessed on 1 September 2019) [31] (PROSITE (https://prosite.expasy.org/; accessed on 1 September 2019) [32], InterProScan (https://www.ebi.ac.uk/interpro/search/sequence/; accessed on 1 September 2019) [33], NCBI (https://www.ncbi.nlm.nih.gov/; accessed on 1 September 2019) [34] and Pfam implemented in HMMER v3.3 (http://hmmer.org/; accessed on 1 September 2019) [35]. Signal peptides were predicted using the online tool SignalP (http://www.cbs.dtu.dk/services/SignalP/; accessed on 1 September 2019) [36]. Predicted domains were visualized using an R script written in-house. 

### 2.4. Comparative Analysis of Conchiferan Shell Matrix Proteins

In order to identify conserved protein sequences among the five Conchiferan species analyzed in this study, the annotated 47 Shell Matrix Protein sequences of *N. pompilius* were used as queries in reciprocal local BLASTx and tBLASTn searches, against the data of the four molluscans for which the Shell Matrix Protein sequence data are already published (71 *Crassostrea gigas* proteins [16]; 159 *Pinctada fucata* proteins [16]; 311 *Lottia gigantea* proteins [12]; 55 *Euhadra quaesita* proteins [4]) (e-value < 1 × 10^−5^ and threshold ≥ 50%: “Search Setting 1”, e-value < 1 × 10^−5^: “Search Setting 2”). The presence of homologous domains was confirmed manually, based on our reciprocal local BLAST result. 

### 2.5. Phylogenetic Analyses of the Shell Matrix Proteins

Phylogenetic analyses were conducted on a total of six Shell Matrix Proteins obtained in this study (Pif/BMSP-like protein, Tyrosinase: Figure 4; CD109 Antigen protein, Chitinase, Peroxidase, EGF-like domain-containing protein: Supplementary Figure S2). In order to do so, homologous amino acid sequences of each protein of various organisms were data-mined from UNIPROT (https://www.uniprot.org/; accessed on 1 September 2019), including molluscan SMPs (if available/when relevant) and non-SMPs. The presence of homologous domains in the sequences was confirmed using HMMER v3.1b2 (http://hmmer.org; accessed on 1 September 2019; e-values < 1 × 10^−5^). These sequences were then aligned using the online version of MAFFT v7.310 (http://mafft.cbrc.jp/alignment/server/index.html; accessed on 1 September 2019; [37]), with the g-INS-i algorithms to allow for global alignment [38]. Sequences were edited using the online version of GBlocks v.091b [39] under the least stringent settings. Model selection was conducted on MEGA v10 [40]. Maximum Likelihood trees were inferred using the GUI version of RAxML [41], with the rapid tree search setting and 1000 bootstrap replications, using the best fitting amino acid substitution model. The selected model for each protein is written directly in the figure legend of each tree. 

## 3. Results

### 3.1. Transcriptomics and Proteomics of the Shell Matrix Proteins in Nautilus pompilius

We conducted transcriptome sequencing of seven pieces (ca. 35 mg each) of the mantle tissue in seven separate runs on an ION-PGM next generation sequencing platform, resulting in about five to six million reads per run. After sequence assembly of all reads from the seven runs combined, 48,633 contigs were obtained, with the largest contig is 13,521 bp-long, the average length of contigs 414 bp and the N50 value 419. Of these, 11,830 contigs (24.3%) encode ORFs longer than 100 amino acid residues, in which 8092 contigs encode for proteins similar to those encoded in the draft genome of the California two-spot octopus (*Octopus* *bimaculoides*), and 3738 encode for non-registered polypeptides, which probably include novel (previously uncharacterized) protein sequences. 

We conducted three runs of the LC-MS/MS mass spectrometer to analyze the extracted total proteins from the shell of a *Nautilus* individual for which the mantle transcriptomes were analyzed. A comparison between obtained protein spectra from the MS/MS analysis and inferred protein spectra of the transcriptome contigs resulted in the identification of 61 proteins. Of these, 14 contigs were not included in further analyses because they contain multiple translation frames, most likely caused by sequencing errors. Annotations of the remaining 47 contigs with single translation frames were conducted by doing BLASTp searches against three different databases: (1) the protein data of *O. bimaculoides* predicted from its genome [42], (2) non-redundant (nr) Genbank sequence database and (3) self-prepared database of known Shell Matrix Proteins (SMPs) (Supplementary Table S1). The annotations were successful in identifying 27 sequences (Table 1).

All assembled Shell Matrix Protein-coding gene sequence data newly acquired in this study are publicly archived on DDBJ/Genbank under the Genbank accession numbers LC653254–LC653300.

### 3.2. Homology Comparisons of the Shell Matrix Proteins and Their Domains among Several Conchiferan Mollusks

**Figure 2:**
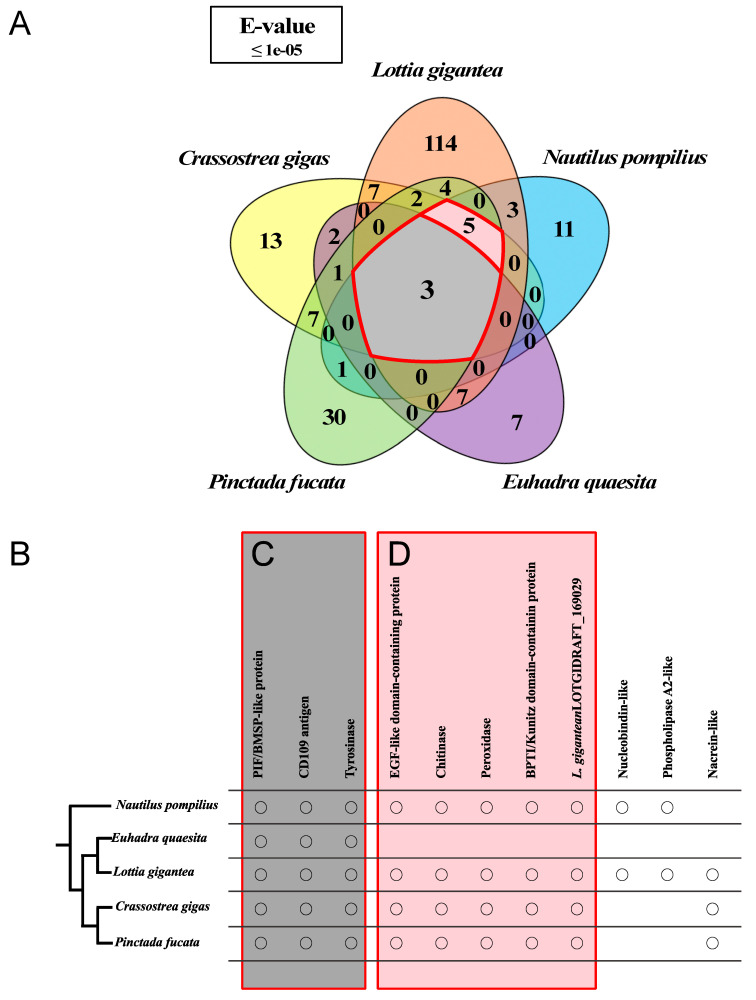
Comparisons of the Shell Matrix Proteins in several Conchiferans for which the data are available using Search Settings 2. Detailed explanation of the settings is written in the main text. (**A**) Venn diagram showing the numbers of shared proteins identified through local BLASTp searches among the five Conchiferans. Red line enclosure is the number of conserved proteins among the Conchifera. (**B**) Homologous proteins of the five Conchiferans compared, plotted on to the phylogeny of the animals. (**C**) Homologous proteins colored deep green were conserved among all five species. (**D**) Homologous proteins colored Pale green were conserved among marine mollusks.

We carried out reciprocal local BLASTn searches among the Shell Matrix Proteins (SMPs) of *Nautilus* and a select set of four Conchiferans for which detailed SMPs data were available as of July 2019 (the pacific oyster *Crassostrea gigas*, the pearl oyster *Pinctada fucata*, the limpet *Lottia gigantea* and the snail *Euhadra quaesita*), in order to identify conserved proteins and protein domains among the SMPs in the Conchiferans. The searches were conducted with the threshold of ≥50% sequence homology, and e-value of ≤1 × 10^−5^ (“Search Setting 1”). Considering the stringency of our searches and the highly frag-mented nature of our transcriptome sequences, there was a possibility that we did not pick up possible conserved protein-coding gene sequences in our data. Therefore, we also conducted reciprocal local BLASTn searches using less stringent settings following previous studies (only by setting the maximum e-value of ≤ 1 × 10^−5^ [4,16], (“Search Setting 2”) (Figure 2).

Reciprocal local BLASTx and tBLASTn searches of the 47 SMP sequences of *Nautilus* as queries under Search Setting 1 found 43 proteins to be specific to *Nautilus* (23 were annotated, while 20 were unknown proteins). However, the less stringent searches found 31 proteins (11 annotated, 20 unknown) to be specific to *Nautilus*. Meanwhile, searches using Search Setting 1 identified no protein, while Search Setting 2 identified additional three proteins (Pif/BMSP-like protein, CD109 Antigen protein, and Tyrosinase) in all Conchiferans. Our most stringent searches identified another protein (EGF-ZP domain-containing protein), and additional four (Chitinase, Peroxidase, Kunitz domain-containing protein and *L. gigantea* LOTGIDRAFT_169029 (Chitin-binding domain-containing protein) by the less stringent searches, to be also shared among the four marine members, excluding *E. quaesita*. Thus, a total of eight proteins (Pif/BMSP-like protein, CD109 Antigen protein, Tyrosinase, Chitinase, Peroxidase, Kunitz domain-containing protein, L. *gigantea* LOTGIDRAFT_169029 and EGF-like domain-containing protein) were found to be conserved among the four marine Conchiferans analyzed in this study (Figure 2B). A complete list of the proteins is shown in Table 1, while the result of reciprocal local BLAST searches is shown in Supplementary Figure S1 and Supplementary Table S2 (for Search Setting 2), and Supplementary Figure S1 and Supplementary Table S3 (Search Setting 1). 

**Figure 3:**
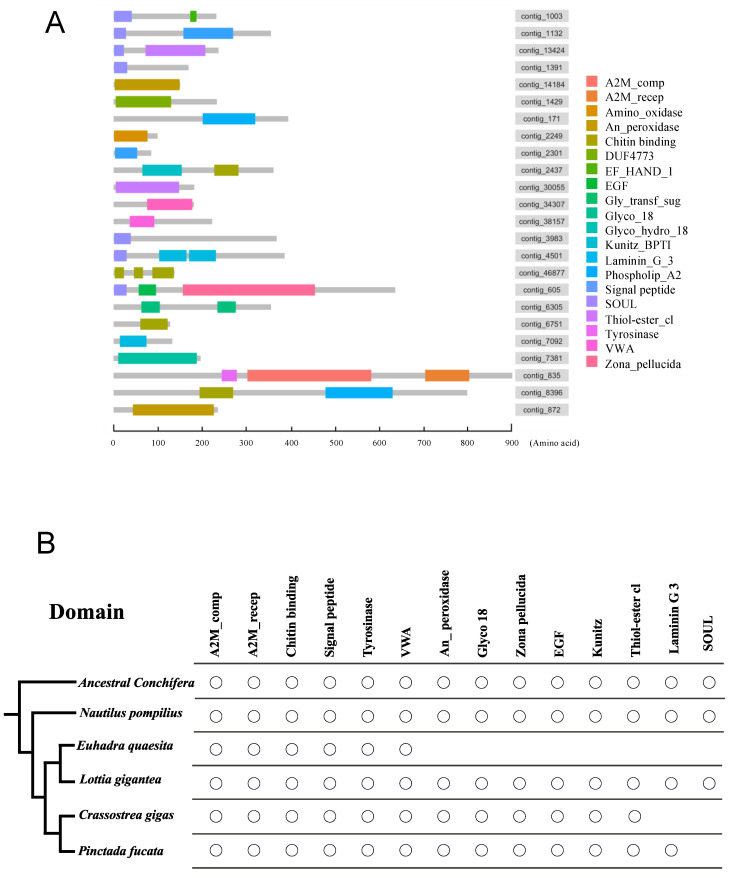
Comparisons of the domains contained in the Shell Matrix Proteins of several Conchiferans for which the data are available. (**A**) Schematic representations of the domains in the Shell Matrix Proteins of *Nautilus pompilius*. (**B**) Shared domains in the Shell Matrix Proteins of the five Conchiferans (*N. pompilius*, *Pinctada* *fucata, Crassostrea gigas, Lottia gigantea* and *Euhadra quaesita*) compared, mapped on to the phylogeny of the animals. The reconstructed Ancestral Conchiferans most likely had all of the shared domains.

**Figure 4:**
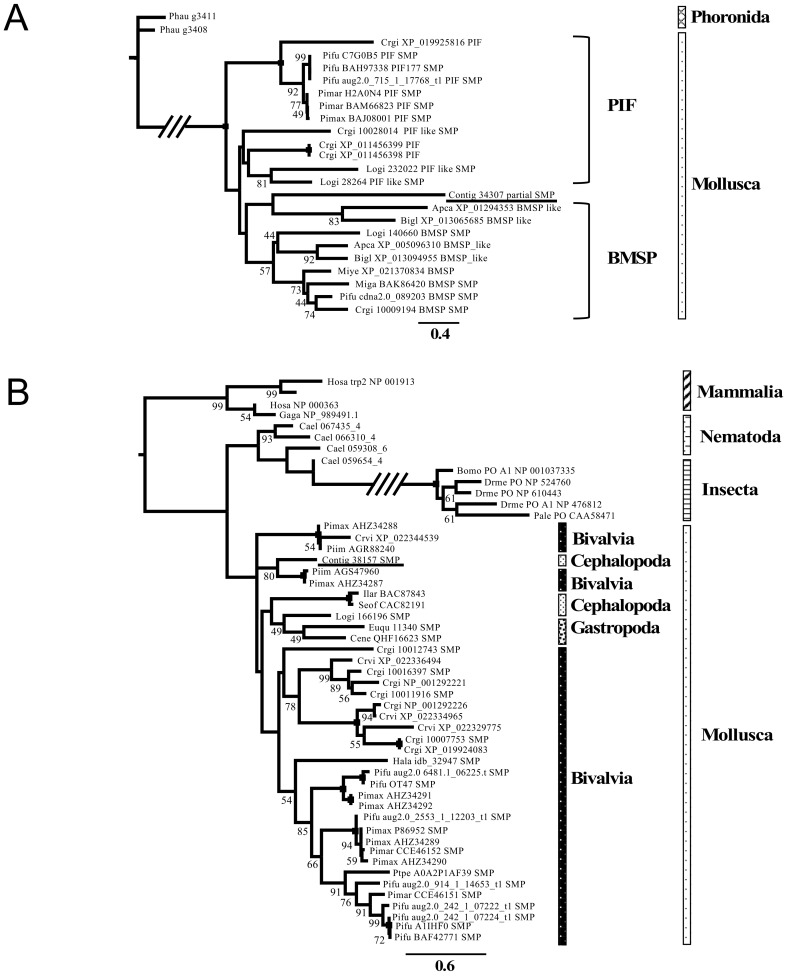
Phylogenetic trees of selected Shell Matrix Proteins. (**A**) The maximum likelihood tree of the Pif/BMSP amino acid sequences, inferred using the LG + Γ model with 1000 bootstrap replicates. (**B**) The maximum likelihood phylogenetic tree of Tyrosinase inferred under the LG + Γ + I model with 1000 bootstrap replicates. Bootstraps values <40% are not shown, and a black square on a node indicates 100% bootstrap support. *Abbreviations*: Apca: *Aplysia californica,* Bigl: *Biomphalaria glabrata*, Bomo: *Bombyx mori*, Cael: *Caenorhabditis elegans*, Cene: *Cepaea nemoralis*, Crgi: *Crassostrea gigas*, Crvi: *Crassostrea virginica*, Drfi: *Drosophila ficusphila*, Drme: *Drosophila melanogaster*, Euqu: *Euhadra quaesita*, Gaga: *Gallus gallus*, Hala: *Haliotis laevigata*, Hosa: *Homo sapiens*, Ilar: *Illex argentines*, Lili: *Littorina littorea*, Logi: *Lottia gigantea*, Miye: *Mizuhopecten yessoensis*, Miga: *Mytilus galloprovincialis*, Mumu: *Mus musculus*, Pale: *Pacifastacus leniusculus*, Phau: *Phoronis australis*, Pimar: *Pinctada margaritifera*, Pimax: *Pinctada maxima*, Ptpe: *Pteria penguin*, Seof: *Sepia officinalis*, Hadi: *Haliotis discus*, Myco: *Mytilus coruscus*, Mytr: *Mytilus trossulus*, Ocvu: *Octopus vulgaris*, Pimarg: *Pinctada margaritifera*, Pifu: *Pinctada fucata*, Piim: *Pinctada imbricata*, Rano: *Rattus norvegicus,* Trps: *Trichinella pseudospiralis*, Toca: *Toxocara canis*. An OTU name started with the word “Contig” denotes the *Nautilus pompilius* sequence obtained in this study.

In-silico domain searches predicted the presence of domains in 22 of the 27 annotated sequences. Meanwhile, of the 20 contigs we were unable to annotate, domains were predicted in one contig. Diagrams showing the domains of the 22 + 1 sequences of *N. pompilius* are shown in Figure 3A and listed in Supplementary Table S3. We manually searched for the presence of the identified domains in the other four Conchiferan Shell Matrix Protein (SMP) datasets. The result was summarized and shown in Figure 4B and Supplementary Tables S4–S7. We found that six domains (A2M_comp, A2M_recep, Chitin-binding Type 2 (ChtBD2), Signal Peptide, Tyrosinase and Von Willebrand Factor Type A (VWA)) were present in the five Conchiferans we analyzed in this study. When the terrestrial gastropod *E. quaesita* was excluded, additional six domains (An_Peroxidase, Glyco_18 domain, Zona Pellucida (ZP), Epidermal Growth Factor-like (EGF), BPTI/Kunitz family of Serine Protease Inhibitors (KU) and Thiol-Ester bond-forming region (Thiol-ester_cl)) were found to be also shared among the four marine Conchiferans (Figure 3B). 

### 3.3. Phylogenetic Analysis of the Shell Matrix Proteins in Conchifera

In order to investigate their molecular evolution, we selected six successfully annotated SMPs (Pif/BMSP-like protein, Tyrosinase: Figure 4; CD109 Antigen protein, Chitinase, Peroxidase, EGF-like domain-containing protein: Supplementary Figure S2), and conducted Maximum Likelihood phylogenetic inferences together with their metazoan homologs which sequences were obtained from GenBank and UniProt. Relatively robust phylogenetic trees were obtained for all six proteins, with most nodes supported moderately to strongly. Deeper nodes were unsupported, despite their general agreement with the accepted metazoan taxonomy. The sequences form monophyletic groups at the phylum level (e.g., Mollusca), but not so at the lower taxonomic levels. However, all analyzed SMPs do not form monophyletic groups with their non-SMP homologs in their consecutive phyla (Figure 4; Supplementary Figure S2).

## 4. Discussion

### 4.1. The Shell of Nautilus pompilius Is a Typical Conchiferan Shell

Like other Conchiferans, the outer shells of the cephalopods are thought to also function by protecting their soft parts against predators. Shell morphological studies have indicated that outer shell breakages caused by fatal and non-fatal predatory attacks were often found in various extant *Nautilus* [43] and extinct, shelled cephalopod fossils [44,45]. Moreover, members of the cephalopods had developed swimming ability, which had assisted their radiation both horizontally and vertically in the ocean habitat, in contrast to the rest of the marine mollusks, which are mostly benthic. Among the shelled cephalopods, such swimming ability was acquired by the formation of chambered shells (outer shell wall + internal septa), which functioned as a hydrostatic apparatus and unique to the cephalopods [46].

Microstructures of the Conchiferan shells have been classified in several ways based on their crystalized mineral morphology and architecture [47]. The differing classification methods however agreed on the presence of the prismatic and nacreous layers, which have been observed in the shell of all Conchiferans including *Nautilus*, various bivalves (e.g., Pterioidea, Mytiloidea and Nuculoidea) and gastropods (e.g., Trochoidea and Haliotoidea). The wide occurrence of these types of microstructures among the Conchiferans strongly suggests that the *Nautilus* shell retains some of the ancestral characters of the Conchiferan shell, and thus most likely, its biomineralization processes.

### 4.2. Homology Comparisons and the Evolution of the Shell Matrix Proteins and Their Domains among Several Conchiferan Mollusks

Homology searches among several Conchiferan mollusks for which the Shell Matrix Proteins (SMPs) have been studied as of July 2019 (the pacific oyster *Crassostrea gigas*, the pearl oyster *Pinctada fucata*, the limpet *Lottia gigantea* and the snail *Euhadra quaesita*) revealed that three proteins (Pif/BMSP-like protein, CD109 Antigen protein and Tyrosinase; Figure 2B) are apparently shared among the Conchiferans. The three proteins are known to be very important in the formation and maintenance of shell structures. For example, the Pif/BMSP proteins are involved in the formation of the nacreous layer of the shell [14,48,49]. Pif and BMSP are composed of signal peptide, von Willebrand Factor Type A domain (VWA), and Chitin-binding domains. The Signal Peptide domain, which function is to guide synthesized proteins to the membrane complex of the cell for secretion, is present in all secretory proteins [50]. The VWA domain is known to function in protein-protein interaction, while Chitin-binding domain is known to interact with calcium ions in calcium carbonate [49]. Tyrosinase (both as a protein and a domain) is known to be involved in pigmentation [51,52], and found in all mollusks compared in this study. The protein was probably recruited to form the diverse coloration and color patterns of the shell. In mammals, including humans, the CD109 Antigen protein is known to be involved in mineralized tissue formation, by being involved in osteoclast formations [53]. Molecularly, it is a protease inhibitor, and it works by regulating TGF-beta receptor expression, TGF-beta signaling and STAT3 activation to inhibit TGF-beta signaling [54,55]. 

In addition to the three proteins detailed above, when the land snail *Euhadra quaesita* was excluded in the reciprocal BLASTx searches, another five proteins (EGF-ZP domain-containing protein, Chitinase, Peroxidase, Kunitz domain-containing protein and *L. gigantea* LOTGIDRAFT_169029 (Chitin-binding domain-containing protein) were found to be conserved among the marine Conchiferans (Figure 3B). While it is very enticing to suggest that the difference in the types of proteins inside the shell matrices were caused by adaptation to terrestrial environment, we cannot conclusively suggest so based only our result reported here, because of the differing sequencing methods and depths of the studies. However, previous reports have suggested that the proteins reported as conserved only among the marine Conchiferans were also probably important during shell formation. For example, the EGF-ZP domain-containing protein, Chitinase and Peroxidase were suggested to be involved in the formation of calcium carbonate crystals in the shell [29,30,56,57,58]. The presence of homologs of these proteins in all Conchiferan SMPs including the basal cephalopod *Nautilus* might have underlined their importance in Conchiferan shell formation.

Two proteins, the Nucleobindin-like and Phospholipase A2-like proteins, were shown to be shared only between the limpet *Lottia gigantea* and *Nautilus*. Nucleobindin is known to be related to calcium ion binding in humans [59]. Phospholipase A2 is a hydrolyzing enzyme which function of cleaving phospholipids depends on the presence of calcium ions [60]. While the specific function of both enzymes during shell formation and biomineralization has never been assessed, we could deduce that both enzymes are probably related to the calcification process of the shell. However, our analyses did not find these two enzymes in the shell matrices of other Conchiferans compared in this study, besides the limpet and *Nautilus*. This could be attributed not only to the exhaustiveness of data, but also to possible evolutionary scenarios, where the two genes were either lost by the other Conchiferan groups, or independently recruited by the limpet and *Nautilus*. Interestingly, the traditional view of molluscan taxonomy puts the gastropods as the sister group of the cephalopods [61,62]. It is also to be noted that we found two Phospholipase A2-like proteins in *Nautilus*.

Based on the information we presently obtained from this study, we could deduce a putative set of Conchiferan SMPs (Figure 2B). However, phylogenetic analyses of the six proteins (Figure 4; Supplementary Figure S2) showed that the SMPs were not monophyletic, as what would be expected if the proteins were specifically recruited as SMPs only once in the ancestral Conchiferan. We found that the SMPs were not monophyletic even among closely related taxa/species (e.g., Tyrosinase: Figure 4B). Therefore, our present findings suggest that the same proteins were probably recruited multiple times in various taxa across Conchiferans from preexisting proteins, which functions and structures were probably useful and easier to tinker for the formation of biomineralized structures. 

From the 47 protein sequences we obtained from the shell of *Nautilus*, we identified the presence of 19 domains (Figure 3A). When compared with other the data of other Conchiferans analyzed in this study, we found that five domains were conserved in all species, five additional domains were conserved only in the marine ones (Figure 4B), and three domains were found only in *Nautilus*. They are common domains usually found in many proteins, including those unrelated to biomineralization in metazoans. However, from our results, we can deduce that the proteins containing these domains were probably recruited for shell formation, because the domains’ known functions indicate that they are most likely related to one or several events of shell formation and maintenance, including biomineralization.

### 4.3. Transcriptomics of the Mantle Tissue in Nautilus pompilius Using ION Torrent PGM Is Arguably Enough to Reveal the Presence of Several Core Shell Matrix Proteins

In this study, we successfully identified 61 Shell Matrix Protein (SMP) sequences, although not all of them were usable in further downstream analyses due to sequencing errors (47 SMPs = without frameshift errors). However, the number of obtained proteins is reasonable, when compared with other previous studies (e.g., *Mya truncata* = 67 [63]; *Crassostrea gigas* = 53 [64]; *Mytilus coruscus* = 63 [65]; *Pinctada fucata* = 75 [66]; *Cepaea nemoralis* = 59 [67]; *Pinctada margaritifera* = 78 [13]; *Euhadra quaesita* = 55 [4]. One of the possible advantages of using an arguably shallower system for transcriptome sequencing (such as ION-PGM) is that most of the sequences we obtained here were probably the most abundantly expressed transcripts (major SMPs), and thus not background expression genes accidentally picked-up. However, using a shallow next generation sequencing system also brings some disadvantages. For example, failure in domain predictions and annotations of several SMP contigs were probably because they were too fragmented and thus the sequences were incomplete, causing annotation programs to fail in detecting any domain. There is also a possibility that sequencing errors might have caused incorrect *in silico*-translations of some contigs. Of course, however, the possibility that some of the contained domains were unpredictable because they were novel domains, and that the 13 protein sequences are novel, previously uncharacterized proteins, cannot be eliminated by our present results. 

For example, in this study, we did not detect the presence of Nautilin-63, which was extracted from the acid-soluble fraction of the shell of a congener of *Nautilus pompilius, N. macromphalus* [68]. This is probably caused by the shallowness of the sequencing system we presently employed in this study, although the possibility that this protein is species specific also cannot be denied. Future analyses are still needed to see if Nautilin-63 is a major protein in all Nautilids, or specific to *N. macromphalus*. We also did not detect the presence of Nacrein, despite its putative crucial role during the formation of nacres in Conchiferan shells [69]. This could probably be attributed to the limitations of the sequencing machine as mentioned above, besides the fact that we only analyzed the water-soluble fraction of the SMPs in this study. Therefore, in order to obtain a more complete picture of SMPs in extant Nautilids, including *N. pompilius*, further studies using deep transcriptome sequencing platforms such as Illumina, and proteomics analyses of both the hydrophilic and hydrophobic components of the SMPs, are still needed in the future.

### 4.4. Concluding Remarks

In this study, we conducted transcriptomics and proteomics analyses of the Shell Matrix Proteins of an extant basally diverging cephalopod, the Nautilid *Nautilus pompilius*. We successfully identified 47 proteins, in which 27 were successfully annotated. We were unable to annotate the other 20 protein sequences probably because they are too short/too fragmented, or because they are previously uncharacterized and/or novel protein sequences. Of the 27 sequences we annotated, we found 11 proteins to be present only in the shell matrix of *Nautilus pompilius* (Table 1). With only our present data, we are unable to actually say if the absence of these proteins in other Conchiferans is biological or technical. This is because the lack of sequence information prohibits us to deduce if the sequences were unique to *Nautilus* or shared with other organisms we compared in this study. For example, it is possible that the protein shared between *Nautilus* and the octopus (hypothetical protein OCBIM_22021924mg [*Octopus bimaculoides*]) is actually a protein sequence specific to the cephalopods, while the heme-binding protein 2-like [*Limulus polyphemus*] is shared between the cephalopods and the limulid horseshoe crab. 

In order to obtain a more in-depth view and conclusive insights regarding the evolution and functions of these proteins during the formation and maintenance of the shell in *Nautilus,* the cephalopods and the Conchiferans, comprehensive future studies involving molecular evolution studies, comparative genomics and functional analyses are needed still needed. For example, studies involving comparison across different taxa will be needed to elucidate the specificity (or non-specificity) of hypothetical protein OCBIM_ 22021924mg [*Octopus bimaculoides*] and heme-binding protein 2-like [*Limulus polyphemus*] in *Nautilus*, while comprehensive functional and molecular evolutionary studies of *L. gigantea* LOTGIDRAFT_169029 should be carried out in order to understand its specific functions during Conchiferan shell formation. 

## Figures and Tables

**Table 1:** Annotation results of the 47 transcriptome contigs, which were identified as Shell Matrix Protein-coding genes by proteome analysis in *Nautilus pompilius*.

Contig ID	FPKM	BLASTn Searches Result	e-Value
contig_130	73.5	None	
contig_145	140,864.70	None	
contig_171	2072.40	Sushi-like protein [*Mytilus coruscus*]	3.00 × 10^−21^
contig_175	2671.90	None	
contig_218	29,476.20	None	
contig_605	321.6	EGF-like domain-containing protein 2 isoform X3 [*Octopus bimaculoides*]	2.00 × 10^−107^
contig_737	41,106.30	None	
contig_749	175,497.30	None	
contig_790	97350.2	None	
contig_835	862.5	CD109 Antigen-like isoform X1 [*Crassostrea gigas*]	0
contig_872	88.1	Chorion Peroxidase-like [*Octopus vulgaris*]	3.00 × 10^−45^
contig_1003	2305.10	protein PFC0760c-like [*Octopus vulgaris*]	1.00 × 10^−3^
contig_1132	1428.80	Phospholipase A2-like [*Centruroides sculpturatus*]	1.00 × 10^−39^
contig_1391	239	hypothetical protein KP79_PYT17609 [*Mizuhopecten yessoensis*]	6.00 × 10^−10^
contig_1429	1.9	None	
contig_2249	6547	Aplysianin-A-like [*Crassostrea virginica*]	9.00 × 10^−6^
contig_2301	77,909.50	hypothetical protein LOTGIDRAFT_176428 [*Lottia gigantea*]	3.00 × 10^−8^
contig_2437	224.1	Chitinase [*Sepia esculenta*]	2.00 × 10^−42^
contig_3214	1694.2	hypothetical protein LOTGIDRAFT_236297 [*Lottia gigantea*]	1.00 × 10^−4^
contig_3983	1112.5	None	
contig_4501	663.3	Papilin-like [*Lingula anatina*]	2.00 × 10^−37^
contig_6305	420.8	uncharacterized protein LOC112560033 isoform X3 [*Pomacea canaliculata*]	2.00 × 10^−24^
contig_6751	2281.00	BMSP [*Mytilus galloprovincialis*]	3.00 × 10^−19^
contig_7092	93.4	Collagen Alpha-3(VI) chain isoform X2 [*Cricetulus griseus*]	6.00 × 10^−8^
contig_7381	440	hypothetical protein OCBIM_22014960mg [*Octopus bimaculoides*]	3.00 × 10^−51^
contig_8396	288.3	Sushi-like protein [*Mytilus coruscus*]	6.00 × 10^−56^
contig_8398	6029.2	None	
contig_11910	1079.4	PREDICTED: nucleobindin-1-like, partial [*Paralichthys olivaceus*]	2.00 × 10^−7^
contig_13424	197.6	Heme-binding protein 2-like [*Limulus polyphemus*]	3.00 × 10^−8^
contig_14184	431.7	Peroxidase-like protein [*Mizuhopecten yessoensis*]	9.00 × 10^−42^
contig_14880	772.8	None	
contig_16223	267.3	None	
contig_17506	164.2	Protein PIF [*Mizuhopecten yessoensis*]	1.00 × 10^−2^
contig_21095	770.1	None	
contig_21964	195.5	None	
contig_23085	71.3	None	
contig_25822	83.8	hypothetical protein KP79_PYT14004 [*Mizuhopecten yessoensis*]	9.00 × 10^−8^
contig_30055	123.8	uncharacterized protein LOC106876168 [*Octopus bimaculoides*]	3.00 × 10^−18^
contig_30170	134.5	Mucin-5AC-like isoform X2 [*Pomacea canaliculata*]	4.00 × 10^−15^
contig_30322	109.8	None	
contig_33774	152.2	None	
contig_34307	12.1	Collagen-like protein-1, partial [*Mytilus coruscus*]	3.00 × 10^−13^
contig_35294	13.7	None	
contig_38157	3.4	Tyrosinase-like protein [*Octopus vulgaris*]	3.00 × 10^−77^
contig_38801	167	None	
contig_46079	0	None	
contig_46877	59.3	hypothetical protein LOTGIDRAFT_169029 [*Lottia gigantea*]	3.00 × 10^−3^

## Data Availability

All assembled sequence data newly acquired in this study are publicly available on DDBJ/Genbank under the Genbank accession numbers LC653254–LC653300.

## Supplementary Materials

The following are available online at https://www.mdpi.com/article/10.3390/genes12121925/s1, Figure S1: Schematic presentation of the homologous relationships of the Shell Matrix Proteins among five Conchiferans, Figure S2: Phylogenetic trees of selected Shell Matrix Proteins, Table S1: Annotation results of the 47 transcriptome contigs, which were identified as shell matrix protein-coding genes by proteome analysis, Table S2: Comparison of Shell Matrix Proteins of four Conchiferans under “Search Setting 1” (sequence homology ≥50%, e-value < 1 × 10^−5^), Table S3: Comparison of Shell Matrix Proteins of four Conchiferans under “Search Setting 2” (e-value < 1 × 10^−5^, Table S4: Annotation results of the 47 transcriptome contigs identified as shell matrix protein-coding genes by proteome analysis, Table S5: The domain of four species (*Pinctada fucata*, *Lottia gigantea*, *Euhadra quaesita* and *Crassostrea gigas*) as predicted by SMART, Table S6: Comparison of the conserved domains among the five species of Conchifera analyzed in this study (*Nautilus pompilius*, *Pinctada fucata*, *Lottia gigantea*, *Euhadra quaesita* and *Crassostrea gigas*), Table S7: The specific domains of the five species of Conchifera analyzed in this study (*Nautilus pompilius*, *Pinctada fucata*, *Lottia gigantea*, *Euhadra quaesita* and *Crassostrea gigas*).
