# Supplementary material for: Hydrophilic Shell Matrix Proteins of Nautilus pompilius and the Identification of a Core Set of Conchiferan Domains
Source: Genes (Basel). 2021 Nov 29;12(12):1925. doi: 10.3390/genes12121925 (PMC8700984; doi:10.3390/genes12121925)
Supplement: Supplementary file 1 [file genes-12-01925-s001.zip › Supp_PDFs/4_Npo_Supp_Fig_Legends.pdf]

Supplementary Figure Legends

Figure S1. Schematic presentation of the homologous relationships of the Shell Matrix Proteins among five Conchiferans (*Pinctada fucata*, *Crassostrea gigas*, *Lottia gigantea*, and *Euhadra quaesita*). These homologous proteins were linked colorfully based on e-value score: Black ≤e-05, >e-10; Blue ≤e-10, >e-20; Red ≤e-20.

Figure S2. Phylogenetic trees of selected Shell Matrix Proteins. (A) The maximum likelihood phylogenetic tree of A2M related CD109 antigen Protein, inferred using the LG + Γ model with 1000 bootstrap replicates. (B) The maximum likelihood phylogenetic tree of Chitinase inferred under the LG + Γ model with 1000 bootstrap replicates. (C) The maximum likelihood tree inferred from Peroxidase amino acid sequences under the LG + Γ model with 1000 bootstrap replicates. (D) The phylogenetic tree of the EGF-ZP Protein under the WAG + Γ model with 1000 bootstrap replicates. Bootstraps values <40% are not shown, and a black square on a node indicates 100% bootstrap support. **Abbreviations**: Apca: *Aplysia californica,* Bigl: *Biomphalaria glabrata*, Bomo: *Bombyx mori*, Cael: *Caenorhabditis elegans*, Cene: *Cepaea nemoralis*, Crgi: *Crassostrea gigas*, Crvi: *Crassostrea virginica*, Drfi: *Drosophila ficusphila*, Drme: *Drosophila melanogaster*, Euqu: *Euhadra quaesita*, Gaga: *Gallus gallus*, Hala: *Haliotis laevigata*, Hosa: *Homo sapiens*, Ilar: *Illex argentines*, Lili: *Littorina littorea*, Logi: *Lottia gigantea*, Miye: *Mizuhopecten yessoensis*, Miga: *Mytilus galloprovincialis*, Mumu: *Mus musculus*, Pale: *Pacifastacus leniusculus*, Phau: *Phoronis australis*, Pimar: *Pinctada margaritifera*, Pimax: *Pinctada maxima*, Ptpe: *Pteria penguin*, Seof: *Sepia officinalis*, Hadi: *Haliotis discus*, Myco: *Mytilus coruscus*, Mytr: *Mytilus trossulus*, Ocvu: *Octopus vulgaris*, Pimarg: *Pinctada margaritifera*, Pifu: *Pinctada fucata*, Piim: Pinctada imbricate, Rano: *Rattus norvegicus,* Trps: *Trichinella pseudospiralis*, Toca: *Toxocara canis*. An OTU name started with the word "Contig_" denotes the *N. pompilius* sequence obtained in this study.
