# Supplementary material for: Hydrophilic Shell Matrix Proteins of Nautilus pompilius and the Identification of a Core Set of Conchiferan Domains
Source: Genes (Basel). 2021 Nov 29;12(12):1925. doi: 10.3390/genes12121925 (PMC8700984; doi:10.3390/genes12121925)
Supplement: Supplementary file 1 [file genes-12-01925-s001.zip › Supp_PDFs/4_Npo_SupplTable3V3.pdf]

Supplementary Table 3. Comparison of Shell Matrix Proteins of four Conchiferans under "Search Setting 2" (e-value ≤e-05)

Nautilus pompilius		*Crassostrea gigas*		*Pinctada fucata*		*Lottia gigantea*		*Euhadra quaesita*		Check
contig_171	uncharacterized protein LOC110461617 [*Mizuhopecten yessoensis*]	CGI_10010359	Asparagine-rich protein	pfu_aug2.0_1358.1_28227.t1	Asparagine-rich protein	Lotgi1|228264	Similar to Pif97/BMSP 1; domains: vWA, chitin-binding PIF	Equ10634	Uncharacterized protein PIF-like	
contig_30322	uncharacterized LOC105326593 precursor [*Crassostrea gigas*]	CGI_10012348	Hemicentin-1	pfu_aug2.0_160.1_00336.t1	Uncharacterized shell protein 26 (Fragment)	Lotgi1|231395	Uncharacterized protein; domains: 2 x chitin-binding peritrophin-A; some similarity to PIF/BMSP 1	Equ14133	Matrilin-like PIF-like	
contig_8396	sushi-like protein [*Mytilus coruscus*]	CGI_10012352	Protein PIF	pfu_aug2.0_219.1_30448.t1	Sushi, von Willebrand factor type A, EGF and pentraxin domain-containing protein 1	Lotgi1|232022	Similar to Pif/BMSP 1; domains: vWA, chitin-binding PIF	Equ15522-15523	Sushi, von Willebrand factor type A, EGF, and pentraxin domain-containing protein	
contig_17506	uncharacterized protein LOC110461617 [*Mizuhopecten yessoensis*]	CGI_10012353	Protein PIF	pfu_aug2.0_7063.1_12916.t1	Shell matrix protein (Fragment)	Lotgi1|237510	Similar to chitin-binding protein P86860 1	Equ21247	Sushi, von Willebrand factor type A, EGF, and pentraxin domain-containing protein	
contig_34307	BMSP [*Mytilus galloprovincialis*]/collagen-like protein-1, partial [*Mytilus coruscus*] PIF	CGI_10028014	Protein PIF-like	pfu_aug2.0_715.1_17768.t1	Protein PIF	Lotgi1|239574	Similar to Pif/BMSP 1; domains: chitin_binding CBM_14/ peritrophin A; Thr-rich motif from aa300-372			
contig_6751	BMSP [*Mytilus galloprovincialis*] PIF	CGI_10004086	Protein PIF-like	pfu_aug2.0_747.1_24365.t1	Uncharacterized	Lotgi1|173138	Similar to BMSP/Pif 1, fragment; domain: CBM_14 (chitin-binding) peritrophin A			PIF/BMSP-like protein
				pfu_aug2.0_747.1_24368.t1	Protein PIF	Lotgi1|140660	Similar to BMSP 1; domains: vWFA			
				pfu_aug2.0_747.1_24369.t1	Uncharacterized	Lotgi1|156525	Uncharacterized protein; domains: CLECT, CUB, Sushi/CCP, LDLRA_2, EGF; pI: 4.6			
				pfu_aug2.0_929.1_31288.t1	Protein PIF					
				pfu_cdna2.0_089203	Collagen alpha-5(VI) chain BMSP					
				pfu_aug2.0_53.1_10184.t1	Shell matrix protein (Fragment)					
				pfu_aug2.0_94.1_13574.t1	Electron transfer flavoprotein-ubiquinone oxidoreductase, mitochondrial					
				pfu_aug2.0_3932.1_09248.t1	Protein PIF					
contig_835	CD109 antigen-like isoform X1 [*Crassostrea gigas*]	CGI_10023767	CD109 antigen	pfu_aug2.0_144.1_13676.t1	CD109 antigen	Lotgi1|229818	Similar to thioester-containing protein/CD109 antigen-like; domains: A2M_N, A2M_N_2	Equ09811	Thioester-containing protein	
		CGI_10023765	CD109 antigen			Lotgi1|211452	Similar thioester-containing protein; α2-macroglobulin family			
						Lotgi1|209261	Similar to thioester-containing protein/α2-macroglobulin			CD109 antigen
						Lotgi1|162872	Similar to thioester-containing protein; domains: α2-macroglobulin			
contig_38157	tyrosinase-like protein [*Octopus vulgaris*]	CGI_10007753	Tyrosinase-like protein 1	pfu_aug2.0_242.1_07222.t1	Tyrosinase-like protein 1	Lotgi1|166196	Similar to tyrosinase 1; 11% Pro; domain: tyrosinase; aa393-462 nine GPPVNP-type repeats	Equ11340	Tyrosinase-like	
		CGI_10011916	Putative tyrosinase-like protein tyr-3	pfu_aug2.0_242.1_07224.t1	Tyrosinase-like protein 1					
		CGI_10012743	Tyrosinase-like protein	pfu_aug2.0_2553.1_12203.t1	Tyrosinase-like protein					Tyrosinase
		CGI_10016397	Histone-lysine N-methyltransferase 2C	pfu_aug2.0_6481.1_06225.t1	Tyrosinase-like protein 1					
				pfu_aug2.0_914.1_14653.t1	Tyrosinase-like protein 1					
				pfu_aug2.0_914.1_14654.t1	Tyrosinase-like protein 1					
contig_2437	chitinase [*Octopus vulgaris*]	CGI_10026605	Chitotriosidase-1 acidic mammalian chitinase isoform X2	pfu_aug2.0_194.1_13762.t1	Putative chitinase 1	Lotgi1|209107	Similar to chitobiase/chitinase			Chitinase
contig_7381	hypothetical protein OCBIM_22014960mg [*Octopus bimaculoides*]			pfu_aug2.0_194.1_13763.t1	Putative chitinase					
contig_14184	Peroxidase-like protein [*Mizuhopecten yessoensis*]	CGI_10017426	Peroxidase-like protein	pfu_aug2.0_1225.1_18190.t1	Uncharacterized	Lotgi1|99791	Uncharacterized protein; domain: An_peroxidase/ Peroxidase_3			
contig_872	Chorion peroxidase [*Crassostrea gigas*]			pfu_aug2.0_14144.1_16516.t1	Proline-rich protein 1	Lotgi1|99809	Uncharacterized protein; domain: An_peroxidase/ Peroxidase_3			
				pfu_aug2.0_2147.1_25317.t1	Peroxidase-like protein	Lotgi1|99852	Uncharacterized protein; domain: An_peroxidase/ Peroxidase_3			Peroxidase
				pfu_aug2.0_2613.1_12224.t1	Peroxidase-like protein					
				pfu_aug2.0_465.1_17456.t1	Peroxidase-like protein					
				pfu_aug2.0_465.1_17459.t1	Peroxidase-like protein					
contig_4501	BPTI/Kunitz domain-containing protein/papilin-like [*Lingula anatina*]	CGI_10015567	KappaPI-actitoxin-Avd3a	pfu_aug2.0_1101.1_04821.t1	Carboxypeptidase inhibitor SmCI	Lotgi1|132911	Similar to Kunitz-type protease inhibitor KCP_HALAI 1			
contig_7092	collagen alpha-4(VI) chain-like isoform X1 [*Acinonyx jubatus*]	CGI_10020756	Chelonianin	pfu_aug2.0_1101.1_04822.t1	BPTI/Kunitz domain-containing protein 2	Lotgi1|113221	Uncharacterized protein/similar to antistasin; domains: antistasin; 15% Cys			
				pfu_aug2.0_1101.1_04823.t1	BPTI/Kunitz domain-containing protein 5	Lotgi1|171918	Similar to antistasin; 17% Cys; domains: antistasin; limited similarity to aa660-950 of lustrin A 1			
				pfu_aug2.0_1638.1_28429.t1	Papilin	Lotgi1|201804	Similar to perlwapin 1; domains: antistasin, WAP, 15% Cys, 11% Pro			KU domian containing protein
				pfu_aug2.0_1638.1_28435.t1	BPTI/Kunitz domain-containing protein 2	Lotgi1|239125	Uncharacterized protein; domains: antistasin, WAP			
				pfu_aug2.0_2907.1_25577.t1	BPTI/Kunitz domain-containing protein 4	Lotgi1|176498	Similar to histone H3			
				pfu_aug2.0_2907.1_25578.t1	BPTI/Kunitz domain-containing protein 4					
				pfu_aug2.0_5814.1_16145.t1	BPTI/Kunitz domain-containing protein 1					
				pfu_aug2.0_729.1_31106.t1	Kunitz-type serine protease inhibitor bitisilin-3 (Fragment)					
				pfu_aug2.0_1101.1_04825.t1	BPTI/Kunitz domain-containing protein 3					
contig_46877	hypothetical protein LOTGIDRAFT_169029 [*Lottia gigantea*]	CGI_10017087	Uncharacterized	pfu_aug2.0_297.1_23818.t1	Uncharacterized	Lotgi1|231869	Uncharacterized protein; domains: chitin-binding peritrophin A; Pro-rich extensin-like			
				pfu_aug2.0_210.1_00425.t1	Uncharacterized	Lotgi1|234405	Uncharacterized protein; domains: chitin-binding, peritrophin A			L. gigantea LOTGIDRAFT_169029 (Chitin binding domain containing protein)
						Lotgi1|160173	Uncharacterized protein; domains: Chitin-binding_2 peritrophin A			
contig_605	Full=EGF-like domain-containing protein 2	CGI_10017543	Gigasin-2	pfu_aug2.0_2116.1_21942.t1	EGF-like domain containing protein 2	Lotgi1|235548	Similar to gigasin-2 1; domains: EGF, ZP_2			
		CGI_10017544	EGF-like domain-containing protein 2	pfu_aug2.0_2116.1_21941.t1	EGF-like domain-containing protein 1 (Fragment)	Lotgi1|235549	Similar to mannose receptor; domains: EGF, CLECT, ZP_2			
		CGI_10017545	EGF-like domain-containing protein 2	pfu_aug2.0_3578.1_29138.t1	EGF-like domain-containing protein 1 (Fragment)	Lotgi1|232718	Uncharacterized protein; domains: EGF; 11% Pro			EGF-ZP domain containing protein
				pfu_aug2.0_495.1_17489.t1	Adhesion G protein-coupled receptor L3					
				pfu_aug2.0_2116.1_21943.t1	EGF-like domain-containing protein 2					
				pfu_aug2.0_838.1_27830.t1	EGF-like domain containing protein 2					

		CGI_10022480	Glioma pathogenesis-related protein 1	pfu_aug2.0_701.1_04487.t2	Peptidase inhibitor 16	Lotgi1|233199	Uncharacterized protein; domains: CAP/VSTPX_like /protease_inhibitor_16			
						Lotgi1|233201	Uncharacterized protein; domains: CAP/allergen V5			SCP domain containing protein (proteinase inhibitar)
						Lotgi1|233200	Uncharacterized protein; domains: CAP/VSTPX_like/protease_inhibitor_16			
		CGI_10003000	Complement C1q-like protein 2	pfu_aug2.0_1919.1_31963.t1	Caprin-2			Equ12964	Sialic acid binding lectin	
				pfu_aug2.0_470.1_00785.t1	Caprin-2			Equ22322	Sialic acid binding lectin	C1Q domain containing protein(Lectin/Caprin-2)
								Equ22329	Complement C1q-like	
		CGI_10007021	Sodium-dependent multivitamin transporter	pfu_aug2.0_862.1_07957.t1	Uncharacterized	Lotgi1|176463	Similar to pacifastin; domains: VWC/pacifastin			
		CGI_10028286	Uncharacterized			Lotgi1|230854	Similar to pacifastin; domains: VWC/pacifastin			Pacifastin
		CGI_10028414	Kielin/chordin-like protein							
		CGI_10014170	Nacrein-like protein (Fragment)	pfu_aug2.0_214.1_13802.t1	Nacrein	Lotgi1|205401	Similar to carbonic anhydrase			
		CGI_10028495	Nacrein-like protein (Fragment)			Lotgi1|238082	Similar to nacrein-like protein 1; domain: α-carbonic anhydrase			Nacrein-like protein
						Lotgi1|239188	aa1-420: similar to nacrein 1; domain: carbonic anhydrase aa421-633: 26% Asp, 23% Gly, 22% Arg, 13% Asn; pI:4.8; similar to aspein 1			
		CGI_10004228	Uncharacterized	pfu_aug2.0_126.1_20287.t1	Midasin	Lotgi1|226726	Uncharacterized protein; domain: chitin_binding_3			
		CGI_10013462	Uncharacterized	pfu_aug2.0_3.1_10035.t1	Uncharacterized					LPMO_10 domain containing protein
		CGI_10018176	Uncharacterized	pfu_aug2.0_39.1_30047.t1	Uncharacterized					
contig_2249	uncharacterized protein LOC110461617 [*Mizuhopecten yessoensis*]			pfu_aug2.0_608.1_27591.t1	Aplysianin-A					Aplysianin-A
contig_2301	hypothetical protein LOTGIDRAFT_176428 [*Lottia gigantea*]					Lotgi1|159173	Uncharacterized protein; domain: partial phospholipase_A2_3			Phospholipase_A2_3
						Lotgi1|176428	Uncharacterized protein; domain: partial Phospholip_A2_3			
contig_30055	uncharacterized protein LOC106876168 [*Octopus bimaculoides*]					Lotgi1|205030	Uncharacterized protein; domain: SOUL			SOUL domain containing protein
contig_11910	nucleobindin-2-like [*Octopus vulgaris*]					Lotgi1|121860	Similar to nucleobindin-2; domains: Efh			Nucleobindin
		CGI_10021817	Vitellogenin-6	pfu_aug2.0_269.1_30539.t1	Apolipophorins					Vitellogenin-6/Apolipophorins
		CGI_10005425	Gigasin-6	pfu_aug2.0_8781.1_06362.t1	Uncharacterized					Gigasin-6
		CGI_10007857	Putative beta-hexosaminidase	pfu_aug2.0_6.1_20028.t1	Putative beta-hexosaminidase					Beta-hexosaminidase
		CGI_10010526	Temptin	pfu_aug2.0_1361.1_04988.t1	DBH-like monooxygenase protein 1					Uncharacterized
		CGI_10015381	Chymotrypsin B	pfu_aug2.0_164.1_13717.t1	Tissue-type plasminogen activator					Chymotrypsin B/Tissue-type plasminogen activator
		CGI_10016430	Uncharacterized shell protein 1	pfu_aug2.0_275.1_17228.t1	Uncharacterized shell protein 1					Uncharacterized
		CGI_10016964	Fibronectin type III domain-containing protein 2	pfu_aug2.0_429.1_30750.t1	Fibronectin type III domain-containing protein 2					
		CGI_10016965	Fibronectin type III domain-containing protein 2	pfu_aug2.0_429.1_30751.t1	Fibronectin type III domain-containing protein 1					Fibronectin type III domain-containing protein
		CGI_10016966	Fibronectin type III domain-containing protein 2	pfu_aug2.0_429.1_30752.t1	Fibronectin type III domain-containing protein 1					
		CGI_10013347	ATP synthase subunit beta, mitochondrial			Lotgi1|201878	Similar to ATP synthase subunit β			ATP synthase subunit β
		CGI_10024501	ATP synthase subunit alpha, mitochondrial			Lotgi1|206617	Similar to ATP synthase subunit α			ATP synthase subunit α
		CGI_10018834	Extracellular superoxide dismutase [Cu-Zn]			Lotgi1|101611	Uncharacterized protein; domain: Cu-Zn superoxide dismutase, fragment			Extracellular superoxide dismutase [Cu-Zn]
		CGI_10008969	L-ascorbate oxidase			Lotgi1|124263	Similar to multicopper oxidase; domains: multicopper oxidase type 1/2			L-ascorbate oxidase/multicopper oxidase
		CGI_10023851	Peptidyl-prolyl cis-trans isomerase B			Lotgi1|222979	Similar to peptidyl-prolyl cis/trans isomerase			Peptidyl-prolyl cis/trans isomerase
						Lotgi1|212757	Similar to peptidyl-prolyl cis/trans isomerase B			
		CGI_10005749	Gigasin-3a (Fragment)					Equ21150	Mesenchyme-specific cell surface glycoprotein	Gigasin-3a/Mesenchyme-specific cell surface glycoprotein
		CGI_10012474	Elongation factor 1-alpha					Equ20990	Elongation factor 1α	Elongation factor 1α
				pfu_aug2.0_2443.1_12165.t1	Poly(U)-specific endoribonuclease	Lotgi1|216792	Similar to si:dkey-222f8.3/endoribonuclease; domain: XendoU			Endoribonuclease
				pfu_aug2.0_490.1_00814.t1	DnaJ homolog subfamily B member 11	Lotgi1|138864	Similar to DnaJ/HSP40; domains: DnaJ, DnaJ_C			DnaJ
				pfu_aug2.0_2922.1_09016.t1	Uncharacterized	Lotgi1|203293	Similar to cAMP-regulated protein-like; domain: cofilin/ADF; N-term: acetyl-Ser			cAMP-regulated protein
				pfu_aug2.0_853.1_11239.t1	non-annotated	Lotgi1|236690	Uncharacterized protein; 22% Gln, 19% Pro; aa268-356: 4 x [xAQPGAYQQP(x)2-4 GAYxQQP]			non-annotated
						Lotgi1|168464	Similar to voltage-dependent anion channel 2-like protein/porin	Equ21047	Voltage-dependent anion-selective channel protein	Voltage-dependent anion-selective channel protein
						Lotgi1|181237	Similar to pleiotrophic membrane chitin-binding protein/chitin deacetylase	Equ21466	Uncharacterized protein	pleiotrophic membrane chitin-binding protein/chitin deacetylase
						Lotgi1|126004	Similar to ubiquitin/polyubiquitin	Equ22616	Ubiquitin-60S ribosomal protein	
						Lotgi1|162671	Similar to ubiquitin/polyubiquitin	Equ32691		Ubiquitin
						Lotgi1|233138	Similar to ubiquitin/polyubiquitin			
						Lotgi1|175997	Similar to histone H2B/H4	Equ53877	Histone H4	Histone H4
						Lotgi1|234386	Uncharacterized protein; 13% Ala, 11% Gly	Equ26417	Hypothetical protein	Uncharacterized
						Lotgi1|234387	Uncharacterized protein			
						Lotgi1|163637	Uncharacterized protein; domain: EFh, 17% Asp, 16%Ala, pI: 3.8; 12 ~30aa repeats	Equ23617-24364	Uncharacterized protein	Uncharacterized
						Lotgi1|193218	Uncharacterized	Equ02505	Beta-actin	
						Lotgi1|202971	Similar to actin; shares peptides with contaminant (bovine actin)	Equ02555	Actin	
						Lotgi1|205506	Similar to actin; shares peptides with contaminant (bovine actin)	Equ04504	Actin	Actin
						Lotgi1|215510	Similar to actin; shares peptides with contaminant (bovine actin)	Equ09762	Beta-actin	
