# Supplementary material for: Hydrophilic Shell Matrix Proteins of Nautilus pompilius and the Identification of a Core Set of Conchiferan Domains
Source: Genes (Basel). 2021 Nov 29;12(12):1925. doi: 10.3390/genes12121925 (PMC8700984; doi:10.3390/genes12121925)
Supplement: Supplementary file 1 [file genes-12-01925-s001.zip › Supp_PDFs/4_Npo_SupplTable1V2.pdf]

Supplementary Table 1. Annotation results of the 47 transcriptome contigs, which were identified as shell matrix protein-coding genes by proteome analysis

contig ID	*O. bimaculoides* homologs	e-value	BLAST against protein	e-value	Local BLASTp against known conchiferan SMPs	e-value
contig_130	None		None		None	
contig_145	None		None		None	
contig_171	Ocbimv22028160m.p	1.92E-23	sushi-like protein [*Mytilus coruscus*]	3.00E-21	Shell matrix protein [*Mizuhopecten yessoensis*]	2.88E-19
contig_175	None		None		None	
contig_218	None		None		None	
contig_605	Ocbimv22007622m.p	7.05E-95	PREDICTED: EGF-like domain-containing protein 2 isoform X3 [*Octopus bimaculoides*]	2.00E-107	Full=EGF-like domain-containing protein 2; AltName: Full=Uncharacterized shell protein 24; Short=LUSP-24; Flags: Precursor [*Lottia gigantea*]	4.32E-36
contig_737	None		None		None	
contig_749	None		None		None	
contig_790	None		None		None	
contig_835	Ocbimv22009422m.p	2.57E-98	CD109 antigen-like isoform X1 [*Crassostrea gigas*]	0	None	
contig_872	Ocbimv22038191m.p	1.17E-47	Chorion peroxidase-like [*Octopus vulgaris*]	3.00E-45	Chorion peroxidase [*Crassostrea gigas*]	6.55E-35
contig_1003	None		protein PFC0760c-like [*Octopus vulgaris*]	1.00E-03	None	
contig_1132	Ocbimv22022244m.p	3.83E-19	phospholipase A2-like [*Centruroides sculpturatus*]	1.00E-39	None	
contig_1391	None		Ahypothetical protein KP79_PYT17609 [*Mizuhopecten yessoensis*]	6.00E-10	None	
contig_1429	None		None		None	
contig_2249	None		aplysianin-A-like [*Crassostrea virginica*]	9.00E-06	None	
contig_2301	None		hypothetical protein LOTGIDRAFT_176428 [*Lottia gigantea*]	3.00E-08	None	
contig_2437	Ocbimv22014960m.p	3.85E-58	Chitinase [*Sepia esculenta*]	2.00E-42	chitinase-3 [*Hyriopsis cumingii*]	1.16E-37
contig_3214	None		hypothetical protein LOTGIDRAFT_236297 [*Lottia gigantea*]	1.00E-04	None	
contig_3983	None		None		None	
contig_4501	Ocbimv22004909m.p	7.33E-15	papilin-like [*Lingula anatina*]	2.00E-37	RecName: Full=BPTI/Kunitz domain-containing protei	1.17E-24
contig_6305	Ocbimv22021904m.p	1.38E-11	uncharacterized protein LOC112560033 isoform X3 [*Pomacea canaliculata*]	2.00E-24	None	
contig_6751	Ocbimv22007257m.p	9.10E-16	BMSP [*Mytilus galloprovincialis*]	3.00E-19	BMSP [*Mytilus galloprovincialis*]	5.83E-25
contig_7092	None		collagen alpha-3(VI) chain isoform X2 [*Cricetulus griseus*]	6.00E-08	nacre serine protease inhibitor 5 [*Pinctada margaritifer*	8.18E-54
contig_7381	Ocbimv22014961m.p	4.32E-55	hypothetical protein OCBIM_22014960mg [*Octopus bimaculoides*]	3.00E-51	Chit3 protein [*Crassostrea gigas*]	8.18E-54
contig_8396	Ocbimv22028160m.p	8.11E-27	Sushi-like protein [*Mytilus coruscus*]	6.00E-56	Shell matrix protein, partial [*Bathymodiolus platifrons*]	5.21E-52
contig_8398			None		None	
contig_11910	Ocbimv22008508m.p	1.10E-12	PREDICTED: nucleobindin-1-like, partial [*Paralichthys olivaceus*]	2.00E-07	None	
contig_13424	None		heme-binding protein 2-like [*Limulus polyphemus*]	3.00E-08	None	
contig_14184	Ocbimv22033454m.p	4.92E-44	Peroxidase-like protein [*Mizuhopecten yessoensis*]	9.00E-42	Chorion peroxidase [*Crassostrea gigas*]	5.56E-44
contig_14880	None		None		None	
contig_16223	None		None		None	
contig_17506	None		Protein PIF [*Mizuhopecten yessoensis*]	1.00E-02	BMSP-like protein [*Lottia gigantea*]	5.85E-08
contig_21095	None		None		None	
contig_21964	None		None		None	
contig_23085	None		None		None	
contig_25822	None		hypothetical protein KP79_PYT14004 [*Mizuhopecten yessoensis*]	9.00E-08	None	
contig_30055	Ocbimv22031901m.p	4.35E-22	uncharacterized protein LOC106876168 [*Octopus bimaculoides*]	3.00E-18	None	
contig_30170	Ocbimv22022137m.p	9.78E-16	mucin-5AC-like isoform X2 [*Pomacea canaliculata*]	4.00E-15	None	
contig_30322	None		None		None	
contig_33774	None		None		None	
contig_34307	Ocbimv22007257m.p	4.58E-15	collagen-like protein-1, partial [*Mytilus coruscus*]	3.00E-13	BMSP [*Mytilus galloprovincialis*]	4.48E-16
contig_35294	None		None		None	
contig_38157	Ocbimv22017648m.p	6.29E-81	tyrosinase-like protein [*Octopus vulgaris*]	3.00E-77	None	
contig_38801	None		None		None	
contig_46079	None		None		None	
contig_46877	None		hypothetical protein LOTGIDRAFT_169029 [*Lottia gigantea*]	3.00E-03	None	
