# Supplementary material for: Hydrophilic Shell Matrix Proteins of Nautilus pompilius and the Identification of a Core Set of Conchiferan Domains
Source: Genes (Basel). 2021 Nov 29;12(12):1925. doi: 10.3390/genes12121925 (PMC8700984; doi:10.3390/genes12121925)
Supplement: Supplementary file 1 [file genes-12-01925-s001.zip › Supp_PDFs/4_Npo_SupplTable5V2.pdf]

Supplementary Table 5. The domain of four species (*Pinctada fucata* , *Lottia gigantea* , *Euhadra quaesita* , and *Crassostrea gigas*) as predicted by SMART

Pinctada fucata		*Lottia gigantea*		*Euhadra quaesita*		*Crassostrea gigas*	
pfu_aug2.0_1101.1_04821.t1	KU	Lotgi1|101611	Sod_Cu	Equ02505	ACTIN	CGI_10003000	C1Q, Signal peptide
pfu_aug2.0_1101.1_04822.t1	KU, H3	Lotgi1|113221	Antistasin	Equ02555	ACTIN	CGI_10004086	VWA, Chitin binding
pfu_aug2.0_1101.1_04823.t1	Signal peptide, KU	Lotgi1|121860	EF-hand_7	Equ04504	ACTIN	CGI_10004228	Signal peptide
pfu_aug2.0_1101.1_04825.t1	KU	Lotgi1|124263	Cu-oxidase, Cu-oxidase_2, Cu-oxidase_3	Equ09762	ACTIN	CGI_10005425	Beta-lactamase, Signal peptide
pfu_aug2.0_1225.1_18190.t1	Signal peptide, SCOP g1cxp.1	Lotgi1|126004	UBQ	Equ09811	A2M_comp, A2M_recep	CGI_10005749	Signal peptide
pfu_aug2.0_126.1_20287.t1		Lotgi1|132911	KU	Equ10634	Chitin binding	CGI_10007021	SSF, VWC
pfu_aug2.0_1358.1_28227.t1	Galactosyl_T	Lotgi1|138864	DnaJ, DnaJ_C	Equ11340	Tyrosinase	CGI_10007753	Tyrosinase
pfu_aug2.0_1361.1_04988.t1	Cu2_monooxygen, Cu2_monoox_C	Lotgi1|140660	VWA	Equ12964	C1Q	CGI_10007857	CHB_HEX, Glyco_hydro_20b, Glyco_hydro_20
pfu_aug2.0_14144.1_16516.t1	An peroxidase, Signal peptide	Lotgi1|151060	DnaJ, Tetratricopeptide repeat-containing	Equ14133	VWA	CGI_10008969	Cu-oxidase, Cu-oxidase_2, Cu-oxidase_3
pfu_aug2.0_144.1_13676.t1	A2M_N, A2M, A2M_recep, A2M_comp, Thiol ester cl	Lotgi1|156525	CLECT, LDLa, CUB, CCP, EGF CA	Equ15522-15523	CCP, Signal petide	CGI_10010359	SCOP d1epwa1
pfu_aug2.0_160.1_00336.t1	Signal peptide	Lotgi1|159173	RPT 1, Phospholip_A2_3	Equ20990	GTP_EFTU	CGI_10010526	Signal peptide
pfu_aug2.0_1638.1_28429.t1	KU	Lotgi1|160173	Chitin binding, Signal peptide	Equ21047	Porin_3	CGI_10011916	Tyrosinase
pfu_aug2.0_1638.1_28435.t1	KU	Lotgi1|162671	UBQ	Equ21150		CGI_10012348	IG, IGc2, Chitin binding
pfu_aug2.0_164.1_13717.t1	Tryp_SPc	Lotgi1|162872	A2M_N, A2M_N_2, A2M, Thiol-ester_cl, A2M_comp, A2M_recep	Equ21247	CCP, Signal petide	CGI_10012352	IGc2, Chitin binding
pfu_aug2.0_1919.1_31963.t1	C1Q, Signal peptide	Lotgi1|163637	SCOP d1gw5a	Equ21466	Polysacc_deac_1	CGI_10012353	EGF, Chitin binding
pfu_aug2.0_194.1_13762.t1	Glyco_18	Lotgi1|166196	Tyrosinase, Signal peptide	Equ22322	C1Q	CGI_10012474	GTP_EFTU, GTP_EFTU_D2, GTP_EFTU_D3
pfu_aug2.0_194.1_13763.t1	Glyco_18, Chitin binding, Signal peptide	Lotgi1|168464	Porin_3	Equ22329	C1Q	CGI_10012743	Tyrosinase
pfu_aug2.0_210.1_00425.t1	Sulfotransfer_2, Chitin binding	Lotgi1|171918	Antistasin, WR1, Signal peptide	Equ22616	UBQ, Ribosomal L40e	CGI_10013347	AAA
pfu_aug2.0_2116.1_21941.t1	ZP, EGF	Lotgi1|173138	Chitin binding	Equ23617-24364	MA	CGI_10013462	LPMO_10, Signal peptide
pfu_aug2.0_2116.1_21942.t1	ZP, EGF, Signal peptide	Lotgi1|175997	H4, H2B	Equ26417		CGI_10014170	Carb_anhydrase, Globin, Signal peptide
pfu_aug2.0_2116.1_21943.t1	ZP	Lotgi1|176428	Phospholip_A2_3	Equ32691	UBQ	CGI_10015381	Tryp_SPc, Signal peptide
pfu_aug2.0_214.1_13802.t1	Carb_anhydrase	Lotgi1|176463	VWC	Equ53877	H4	CGI_10015567	KU
pfu_aug2.0_2147.1_25317.t1	An peroxidase, Signal peptide	Lotgi1|176498	H3			CGI_10016397	Tyrosinase, AT_hook, PHD, RING, PDB 2YUK|A, Glyco_hydro_9
pfu_aug2.0_219.1_30448.t1	VWA, CCP, Chirin binding, Signal peptide	Lotgi1|181237	Polysacc_deac_1, Signal peptide			CGI_10016430	
pfu_aug2.0_242.1_07222.t1	Tyrosinase, Signal peptide	Lotgi1|193218	ACTIN			CGI_10016964	FN3, Signal peptide
pfu_aug2.0_242.1_07224.t1	Tyrosinase, Signal peptide	Lotgi1|201804	WAP, Antistasin, Lustrin_cystein, WR1			CGI_10016965	FN3
pfu_aug2.0_2443.1_12165.t1	PDB 2C1W|C, SO	Lotgi1|201878	ATP-synt_ab_N, AAA			CGI_10016966	FN3
pfu_aug2.0_2553.1_12203.t1	Tyrosinase	Lotgi1|202971	ACTIN			CGI_10017087	Chitin binding
pfu_aug2.0_2613.1_12224.t1	An peroxidase	Lotgi1|203293	ADF			CGI_10017426	An_peroxidase
pfu_aug2.0_269.1_30539.t1	VWD, DUF1943, LPD_N	Lotgi1|205030	SOUL, Signal peptide			CGI_10017543	ZP, EGF, Signal peptide
pfu_aug2.0_275.1_17228.t1		Lotgi1|205401	Carb_anhydrase			CGI_10017544	ZP, EGF, Signal peptide
pfu_aug2.0_2907.1_25577.t1	KU, Antistasin, Signal peptide	Lotgi1|205506	ACTIN			CGI_10017545	ZP, EGF, Signal peptide
pfu_aug2.0_2907.1_25578.t1	KU, Antistasin	Lotgi1|206617	ATP-synt_ab_N, ATP-synt_ab, ATP-synt_ab_C			CGI_10018176	LPMO_10, Signal peptide
pfu_aug2.0_2922.1_09016.t1	ADF	Lotgi1|209107	Glyco_18, Signal peptide			CGI_10018834	Sod_Cu, Signal peptide
pfu_aug2.0_297.1_23818.t1	Chirin binding, Signal peptide	Lotgi1|209261	A2M_N, A2M_N_2, A2M, Thiol-ester_cl, A2M_comp, A2M recep			CGI_10020756	KU
pfu_aug2.0_3.1_10035.t1	LPMO_10, Signal peptide	Lotgi1|211452	A2M, Thiol-ester_cl, A2M_comp, A2M recep			CGI_10021817	LPD_N, DUF1943, VWD, Signal peptide
pfu_aug2.0_3578.1_29138.t1	ZP, EGF	Lotgi1|212757	Pro_isomerase, Signal peptide			CGI_10022480	SCP
pfu_aug2.0_39.1_30047.t1	LPMO_10	Lotgi1|215510	ACTIN			CGI_10023765	A2M, Thiol-ester_cl, A2M_comp, A2M_recep
pfu_aug2.0_3932.1_09248.t1	VWA, Chirin binding, Signal peptide	Lotgi1|216792	XendoU			CGI_10023767	A2M_N, A2M_N_2
pfu_aug2.0_429.1_30750.t1	FN3	Lotgi1|222979	Pro_isomerase, Signal peptide			CGI_10023851	Pro_isomerase
pfu_aug2.0_429.1_30751.t1	FN3	Lotgi1|226726	LPMO_10, Signal peptide			CGI_10024501	ATP-synt_ab_N, ATP-synt_ab, ATP-synt_ab_C
pfu_aug2.0_429.1_30752.t1	FN3, SCOP d1qg3a1, Signal peptide	Lotgi1|228264	VWA, Chitin binding, Signal peptide			CGI_10026605	Glyco_18, Chitin binding
pfu_aug2.0_465.1_17456.t1	An peroxidase	Lotgi1|229818	A2M_N, A2M_N_2, Signal peptide			CGI_10028014	VWA, Chitin binding, Signal peptide
pfu_aug2.0_465.1_17459.t1	An peroxidase	Lotgi1|230854	VWC			CGI_10028286	WR1, VWC
pfu_aug2.0_470.1_00785.t1	C1Q, Signal peptide	Lotgi1|231395	SCOP d1c4ra_, Chitin binding, Signal peptide			CGI_10028414	VWC
pfu_aug2.0_490.1_00814.t1	DnaJ_C, DnaJ, Signal peptide	Lotgi1|231869	Chitin binding			CGI_10028495	Carb_anhydrase, Signal peptide
pfu_aug2.0_495.1_17489.t1	CLECT, HormR, GAIN, GPS, 7tm_2	Lotgi1|232022	VWA, Chitin binding				
pfu_aug2.0_53.1_10184.t1	Laminin_G_3, Chitin binding	Lotgi1|232718	EGF, Signal peptide				
pfu_aug2.0_5814.1_16145.t1	KU, Signal peptide	Lotgi1|233138	UBQ				
pfu_aug2.0_6.1_20028.t1	CHB_HEX, Glyco_hydro_20b, Glyco_hydro_20	Lotgi1|233199	SCP, Signal peptide				
pfu_aug2.0_608.1_27591.t1	Amino_oxidase, NAD_binding_9	Lotgi1|233200	SCP, Signal peptide				
pfu_aug2.0_6481.1_06225.t1	Tyrosinase, Signal peptide	Lotgi1|233201	SCP, Signal peptide				
pfu_aug2.0_701.1_04487.t2	Sh KT, SCP	Lotgi1|234386	Signal peptide				
pfu_aug2.0_7063.1_12916.t1	Laminin_G_3, Chitin binding	Lotgi1|234387					
pfu_aug2.0_715.1_17768.t1	VWA, Chitin binding, Signal peptide	Lotgi1|234405	Chitin binding, Signal peptide				
pfu_aug2.0_729.1_31106.t1	KU	Lotgi1|234561	UBQ				
pfu_aug2.0_747.1_24365.t1	Chitin binding	Lotgi1|235548	EGF, ZP, Signal peptide				
pfu_aug2.0_747.1_24368.t1	Chitin binding	Lotgi1|235549	EGF, ZP, CLECT, Sh KT				
pfu_aug2.0_747.1_24369.t1	SCOP d1c4ra, EGF, Chitin binding	Lotgi1|236690	Signal peptide				
pfu_aug2.0_838.1_27830.t1	ZP, Signal peptide	Lotgi1|237510	Laminin_G_3				
pfu_aug2.0_853.1_11239.t1	Signal peptide	Lotgi1|238082	Carb_anhydrase				
pfu_aug2.0_862.1_07957.t1	WR1	Lotgi1|239125	WAP, WR1, Antistasin, Signal peptide				
pfu_aug2.0_8781.1_06362.t1	Beta-lactamase	Lotgi1|239188	Carb_anhydrase, Signal peptide				
pfu_aug2.0_914.1_14653.t1	Tyrosinase	Lotgi1|239574	Chitin binding, Signal peptide				
pfu_aug2.0_914.1_14654.t1	Tyrosinase	Lotgi1|99791	An_peroxidase				
pfu_aug2.0_929.1_31288.t1	Chitin binding	Lotgi1|99809	An_peroxidase				
pfu_aug2.0_94.1_13574.t1	Thi4, ETF_QO, FAD_binding_2, Chitin binding	Lotgi1|99852	An_peroxidase				
pfu_cdna2.0_089203	VWA, Chitin binding, Signal peptide						
