# Supplementary material for: Hydrophilic Shell Matrix Proteins of Nautilus pompilius and the Identification of a Core Set of Conchiferan Domains
Source: Genes (Basel). 2021 Nov 29;12(12):1925. doi: 10.3390/genes12121925 (PMC8700984; doi:10.3390/genes12121925)
Supplement: Supplementary file 1 [file genes-12-01925-s001.zip › Supp_PDFs/4_Npo_SupplTable7V2.pdf]

Supplementary Table 7. The specific domains of the five species of Conchifera analyzed in this study (*Nautilus pompilius*, *Pinctada fucata*, *Lottia gigantea*, *Euhadra quaesita*, and *Crassostrea gigas*)

Npo	Pfu	Lgi	Equ	Cgi
Glyco_hydro_18	Cu2_monoox_C	CUB	MA	AT_hook
	7tm_2	EF-hand_7	Ribosomal L40e	Globin
	Cu2_monooxygen	EGF CA		Glyco_hydro_9
	ETF_QO	H2B		GTP_EFTU_D2
	FAD_binding_2	LDLa		GTP_EFTU_D3
	GAIN	Lustrin_cystein		IG
	Galactosyl_T	RPT 1		IGc2
	GPS	SCOP d1c4ra_		PDB 2YUK|A
	HormR	SCOP d1gw5a		PHD
	NAD_binding_9	Tetratricopeptide repeat-containing domain		RING
	PDB 2C1W|C			SCOP d1epwa1
	SCOP d1c4ra			SSF
	SCOP d1qg3a1			
	SCOP g1cxp.1			
	SO			
	Sulfotransfer_2			
	Thi4			
