# Supplementary material for: Hydrophilic Shell Matrix Proteins of Nautilus pompilius and the Identification of a Core Set of Conchiferan Domains
Source: Genes (Basel). 2021 Nov 29;12(12):1925. doi: 10.3390/genes12121925 (PMC8700984; doi:10.3390/genes12121925)
Supplement: Supplementary file 1 [file genes-12-01925-s001.zip › Supp_PDFs/4_Npo_SupplTable2V3.pdf]

Supplementary Table 2. Comparison of Shell Matrix Proteins of four Conchiferans under "Search Setting 1" (sequence homology ≥50%, e-value ≤e-05)

<table>
<tr><th colspan="2">Nautilus pompilius</th><th colspan="2">Lottia gigantea</th><th colspan="2">Euhadra quaesita</th><th colspan="2">Euhadra quaesita</th><th colspan="2">Pinctada fucata</th><th></th></tr>
<tr><td>contig_14184</td><td>Peroxidase-like protein [Mizuhopecten yessoensis]</td><td>Lotgi1|99791</td><td>Uncharacterized protein; domain: An_peroxidase/ Peroxidase_3</td><td></td><td></td><td></td><td></td><td></td><td></td><td rowspan="3">Peroxidase</td></tr>
<tr><td>contig_14184</td><td>Peroxidase-like protein [Mizuhopecten yessoensis]</td><td>Lotgi1|99809</td><td>Uncharacterized protein; domain: An_peroxidase/ Peroxidase_3</td><td></td><td></td><td></td><td></td><td></td><td></td></tr>
<tr><td>contig_14184</td><td>Peroxidase-like protein [Mizuhopecten yessoensis]</td><td>Lotgi1|99852</td><td>Uncharacterized protein; domain: An_peroxidase/ Peroxidase_3</td><td></td><td></td><td></td><td></td><td></td><td></td></tr>
<tr><td>contig_30055</td><td>uncharacterized protein LOC106876168 [Octopus bimaculoides]</td><td>Lotgi1|205030</td><td>Uncharacterized protein; domain: SOUL</td><td></td><td></td><td></td><td></td><td></td><td></td><td>SOUL containing protein</td></tr>
<tr><td>contig_605</td><td>Full=EGF-like domain-containing protein 2</td><td>Lotgi1|235548</td><td>Similar to gigasin-2 1; domains: EGF, ZP_2</td><td>CGI_10017543</td><td>Gigasin-2</td><td></td><td></td><td>pfu_aug2.0_2116.1_21941.t1</td><td>EGF-like domain-containing protein 1 (Fragment)</td><td rowspan="5">EGF-ZP domain containing protein</td></tr>
<tr><td></td><td></td><td></td><td></td><td>CGI_10017544</td><td>EGF-like domain-containing protein 2</td><td></td><td></td><td>pfu_aug2.0_2116.1_21942.t1</td><td>EGF-like domain containing protein 2</td></tr>
<tr><td></td><td></td><td></td><td></td><td>CGI_10017545</td><td>EGF-like domain-containing protein 2</td><td></td><td></td><td>pfu_aug2.0_2116.1_21943.t1</td><td>EGF-like domain-containing protein 2</td></tr>
<tr><td></td><td></td><td></td><td></td><td></td><td></td><td></td><td></td><td>pfu_aug2.0_3578.1_29138.t1</td><td>EGF-like domain-containing protein 1 (Fragment)</td></tr>
<tr><td></td><td></td><td></td><td></td><td></td><td></td><td></td><td></td><td>pfu_aug2.0_838.1_27830.t1</td><td>EGF-like domain containing protein 2</td></tr>
<tr><td>contig_835</td><td>CD109 antigen-like isoform X1 [Crassostrea gigas]</td><td>Lotgi1|162872</td><td>Similar to thioester-containing protein; domains: α2-macroglobulin</td><td>CGI_10023765</td><td>CD109 antigen</td><td>Equ09811</td><td>Thioester-containing protein</td><td></td><td></td><td rowspan="2">CD109 antigen</td></tr>
<tr><td></td><td></td><td>Lotgi1|211452</td><td>Similar thioester-containing protein; α 2-macroglobulin family</td><td></td><td></td><td></td><td></td><td></td><td></td></tr>
<tr><td>contig_8396</td><td>sushi-like protein [Mytilus coruscus]</td><td>Lotgi1|228264</td><td>Similar to Pif97/BMSP 1; domains: vWA, chitin-binding</td><td>CGI_10012353</td><td>Protein PIF</td><td>Equ10634</td><td>Uncharacterized protein</td><td>pfu_aug2.0_7063.1_12916.t1</td><td>Shell matrix protein (Fragment)</td><td rowspan="5">PIF/BMSP-like protein</td></tr>
<tr><td>contig_171</td><td>uncharacterized protein LOC110461617 [Mizuhopecten yessoensis]</td><td>Lotgi1|232022</td><td>Similar to Pif/BMSP 1; domains: vWA, chitin-binding</td><td></td><td></td><td></td><td></td><td>pfu_aug2.0_160.1_00336.t1</td><td>Uncharacterized shell protein 26 (Fragment)</td></tr>
<tr><td></td><td></td><td>Lotgi1|231395</td><td>Uncharacterized protein; domains: 2 x chitin-binding peritrophin-A; some similarity to PIF/BMSP 1</td><td></td><td></td><td></td><td></td><td>pfu_aug2.0_747.1_24369.t1</td><td>Uncharacterized protein</td></tr>
<tr><td></td><td></td><td>Lotgi1|237510</td><td>Similar to chitin-binding protein P86860 1</td><td></td><td></td><td></td><td></td><td>pfu_aug2.0_929.1_31288.t1</td><td>Protein PIF</td></tr>
<tr><td></td><td></td><td></td><td></td><td></td><td></td><td></td><td></td><td>pfu_aug2.0_715.1_17768.t1</td><td>Protein PIF</td></tr>
</table>
