# Supplementary material for: Hydrophilic Shell Matrix Proteins of Nautilus pompilius and the Identification of a Core Set of Conchiferan Domains
Source: Genes (Basel). 2021 Nov 29;12(12):1925. doi: 10.3390/genes12121925 (PMC8700984; doi:10.3390/genes12121925)
Supplement: Supplementary file 1 [file genes-12-01925-s001.zip › Supp_PDFs/4_Npo_SupplTable6V2.pdf]

Supplementary Table 6. Comparison of the conserved domains among the five species of Conchifera analyzed in this study (*Nautilus pompilius, Pinctada fucata*, *Lottia gigantea*, *Euhadra quaesita*, and *Crassostrea gigas*)

N. po	*P. fu*	*L. gi*	*E. qu*	*C. gi*
A2M_comp	A2M_comp	A2M_comp	A2M_comp	A2M_comp
A2M_recep	A2M_recep	A2M_recep	A2M_recep	A2M_recep
Chitin binding	Chitin binding	Chitin binding	Chitin binding	Chitin binding
signal peptide	signal peptide	signal peptide	signal peptide	signal peptide
Tyrosinase	Tyrosinase	Tyrosinase	Tyrosinase	Tyrosinase
VWA	VWA	VWA	VWA	VWA
ZP	ZP	ZP		ZP
KU	KU	KU		KU
EGF	EGF	EGF		EGF
An_peroxidase	An_peroxidase	An_peroxidase		An_peroxidase
Glyco_18	Glyco_18	Glyco_18		Glyco_18
Thiol-ester_cl	Thiol ester cl	Thiol-ester_cl		Thiol-ester_cl
Laminin_G_3	Laminin_G_3	Laminin_G_3		
Amino_oxidase	Amino_oxidase			
Phospholip_A2_3		Phospholip_A2_3		
SOUL		SOUL		
	CCP	CCP	CCP	
	A2M	A2M		A2M
	A2M_N	A2M_N		A2M_N
	Carb_anhydrase	Carb_anhydrase		Carb_anhydrase
	LPMO_10	LPMO_10		LPMO_10
	SCP	SCP		SCP
	WR1	WR1		WR1
	C1Q		C1Q	C1Q
	ADF	ADF		
	Antistasin	Antistasin		
	CLECT	CLECT		
	DnaJ	DnaJ		
	DnaJ_C	DnaJ_C		
	H3	H3		
	Sh KT	Sh KT		
	Beta-lactamase			Beta-lactamase
	CHB_HEX			CHB_HEX
	DUF1943			DUF1943
	FN3			FN3
	Glyco_hydro_20			Glyco_hydro_20
	Glyco_hydro_20b			Glyco_hydro_20b
	LPD_N			LPD_N
	Tryp_SPc			Tryp_SPc
	VWD			VWD
		ACTIN	ACTIN	
		H4	H4	
		Polysacc_deac_1	Polysacc_deac_1	
		Porin_3	Porin_3	
		UBQ	UBQ	
		A2M_N_2		A2M_N_2
		AAA		AAA
		ATP-synt_ab		ATP-synt_ab
		ATP-synt_ab_C		ATP-synt_ab_C
		ATP-synt_ab_N		ATP-synt_ab_N
		Cu-oxidase		Cu-oxidase
		Cu-oxidase_2		Cu-oxidase_2
		Cu-oxidase_3		Cu-oxidase_3
		Pro_isomerase		Pro_isomerase
		Sod_Cu		Sod_Cu
		VWC		VWC
			GTP_EFTU	GTP_EFTU
