# Supplementary material for: Hydrophilic Shell Matrix Proteins of Nautilus pompilius and the Identification of a Core Set of Conchiferan Domains
Source: Genes (Basel). 2021 Nov 29;12(12):1925. doi: 10.3390/genes12121925 (PMC8700984; doi:10.3390/genes12121925)
Supplement: Supplementary file 1 [file genes-12-01925-s001.zip › Supp_PDFs/4_Npo_SupplTable4V2.pdf]

Supplementary Table 4. Annotation results of the 47 transcriptome contigs identified as shell matrix protein-coding genes by proteome analysis

ID	Domain				
	SMART	PROSITE	NCBI	InterProScan	Domain Conclusion
contig_11910					
contig_14184	Pfam:An_peroxidase	PEROXIDASE_3	peroxinectin_like<br>An_peroxidase<br>PLN02283		An_peroxidase
contig_171			Laminin_G_3		Laminin_G_3
contig_17506					
contig_2249			Amino_oxidase		Amino_oxidase
contig_2301			Phospholip_A2_3		Phospholipase A2
contig_2437	Pfam:Glyco_hydro_18<br>ChtBD2	CHIT_BIND_II	GH18_chitolectin_chitotriosidase<br>Glyco_18<br>CBM_14<br>ChtBD2<br>Glyco_hydro_18	Glycoside hydrolase family 18, catalytic domain<br>Chitin binding domain	Glyco_hydro_18<br>Chitin binding
contig_30055	Pfam:SOUL		SOUL		SOUL
contig_30322					
contig_34307	Pfam:VWA_2<br>Pfam:VWA	VWFA	VWA<br>vWFA_subfamily_ECM<br>ChlD	von Willebrand factor, type A	von Willebrand factor, type A
contig_38157	Pfam:Tyrosinase		Tyrosinase		Tyrosinase
contig_4501	signal peptide<br>KU<br>transmembrane region	BPTI_KUNITZ_1<br>BPTI_KUNITZ_2	Kunitz_BPTI<br>KU	Pancreatic trypsin inhibitor Kunitz domain	signal peptide<br>Kunitz
contig_46877	Pfam:CBM_14	CHIT_BIND_II			Chitin binding
contig_605	transmembrane region<br>EGF<br>ZP	EGF_3<br>ZP_2<br>EGF_1	ZP<br>Zona_pellucida		Zona_pellucida<br>EGF
contig_6751	ChtBD2	CHIT_BIND_II	ChtBD2<br>CBM_14	Chitin binding domain	Chitin binding
contig_7092	KU	BPTI_KUNITZ_2	KU<br>Kunitz_BPTI	Pancreatic trypsin inhibitor Kunitz domain	Kunitz
contig_7381	Glyco_18		GH18_chitolectin_chitotriosidase<br>Glyco_18<br>Glyco_hydro_18<br>ChiA	Glycoside hydrolase family 18, catalytic domain<br>Chitinase II	Glyco_18
contig_835	Thiol-ester_cl<br>Pfam:A2M_comp<br>A2M_recep		A2M_2<br>A2M_comp<br>A2M_recep<br>YfaS		A2M_comp<br>A2M_recep<br>Thiol-ester_cl
contig_8396	ChtBD2	CHIT_BIND_II		Chitin binding domain	Chitin binding
contig_872	Pfam:An_peroxidase	PEROXIDASE_3	peroxinectin_like<br>An_peroxidase<br>PLN02283		An_peroxidase
