# Supplementary figures and images for: Hydrophilic Shell Matrix Proteins of Nautilus pompilius and the Identification of a Core Set of Conchiferan Domains
Source: Genes (Basel). 2021 Nov 29;12(12):1925. doi: 10.3390/genes12121925 (PMC8700984; doi:10.3390/genes12121925)

Supplementary Fig. 2

A

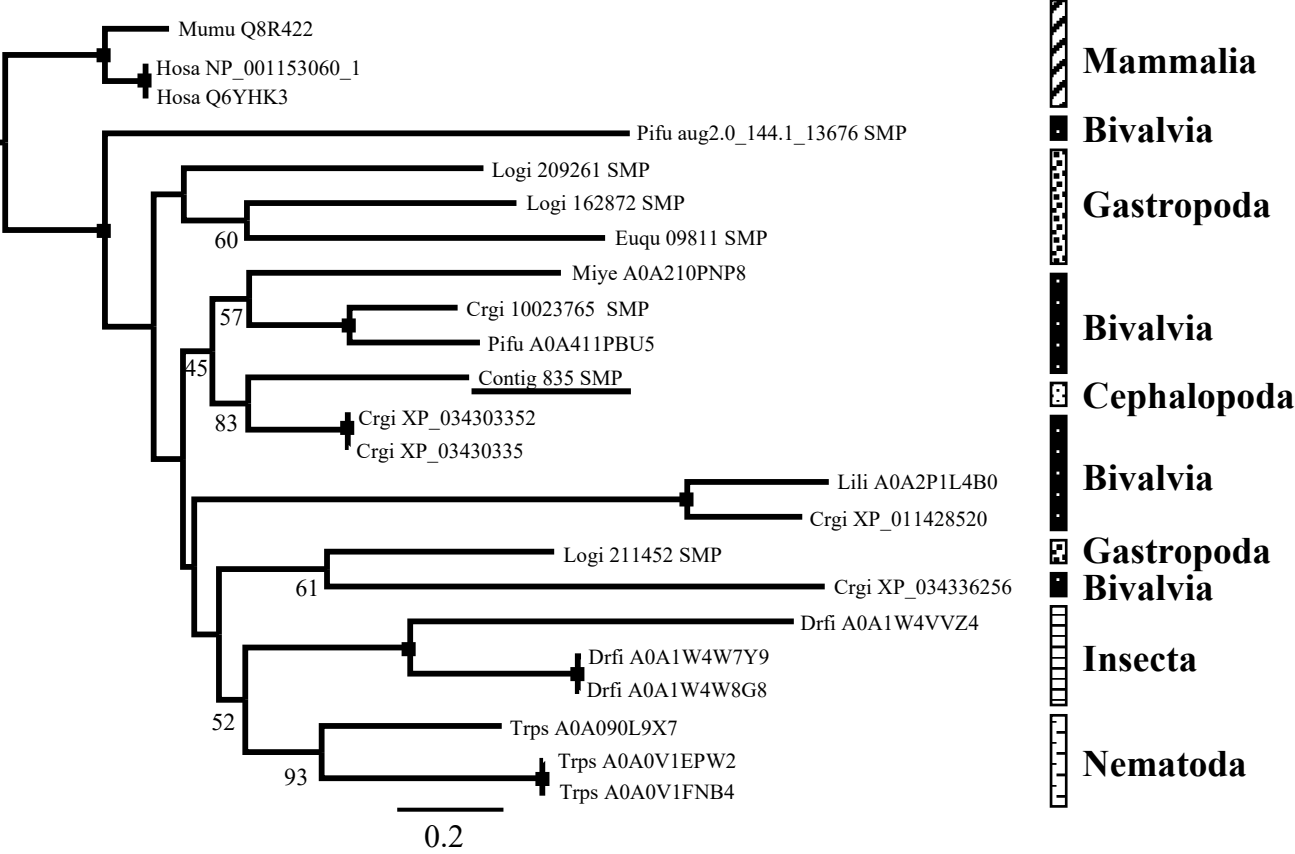

Supplementary Fig. 2

B

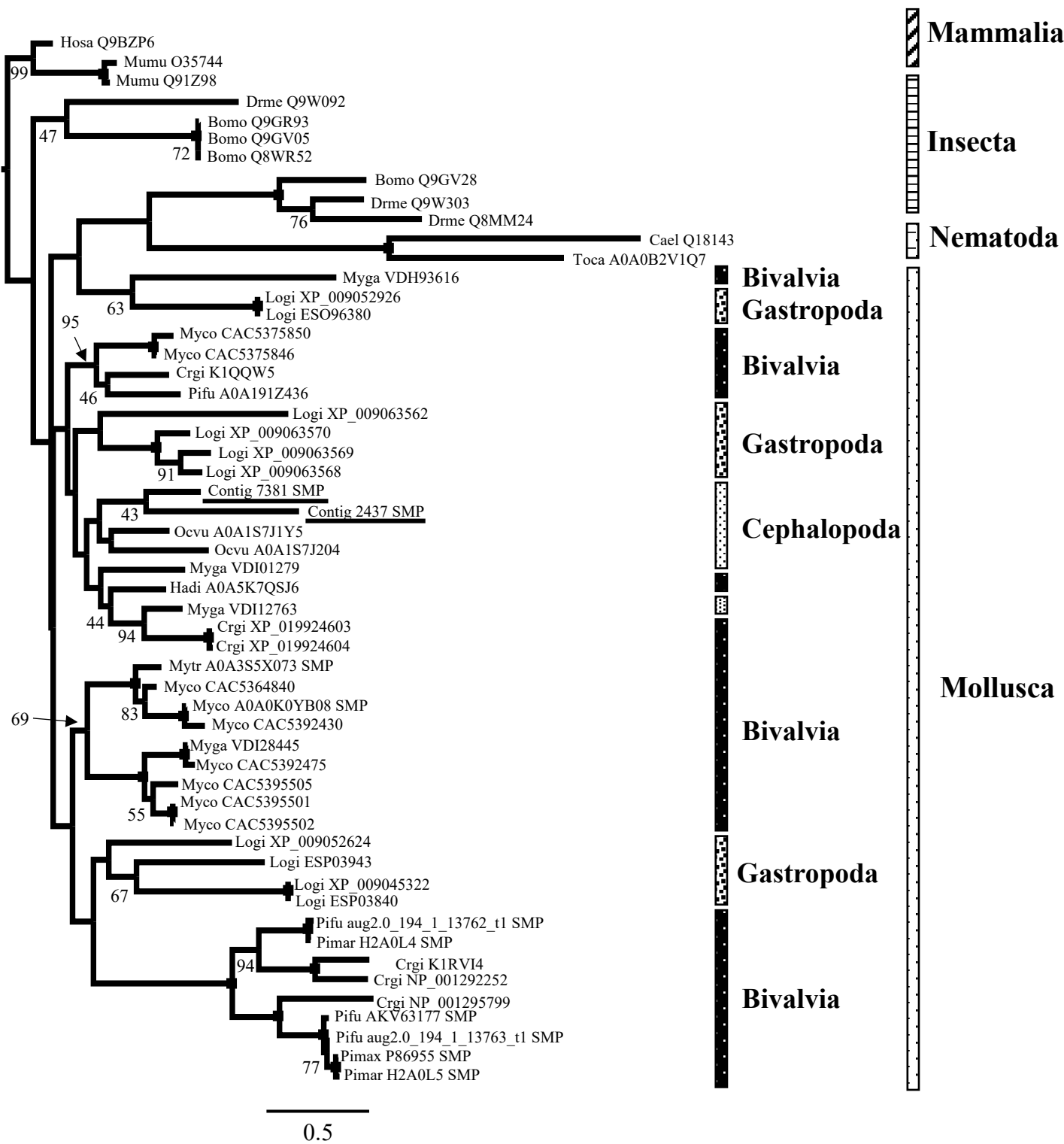

Supplementary Fig. 2

C

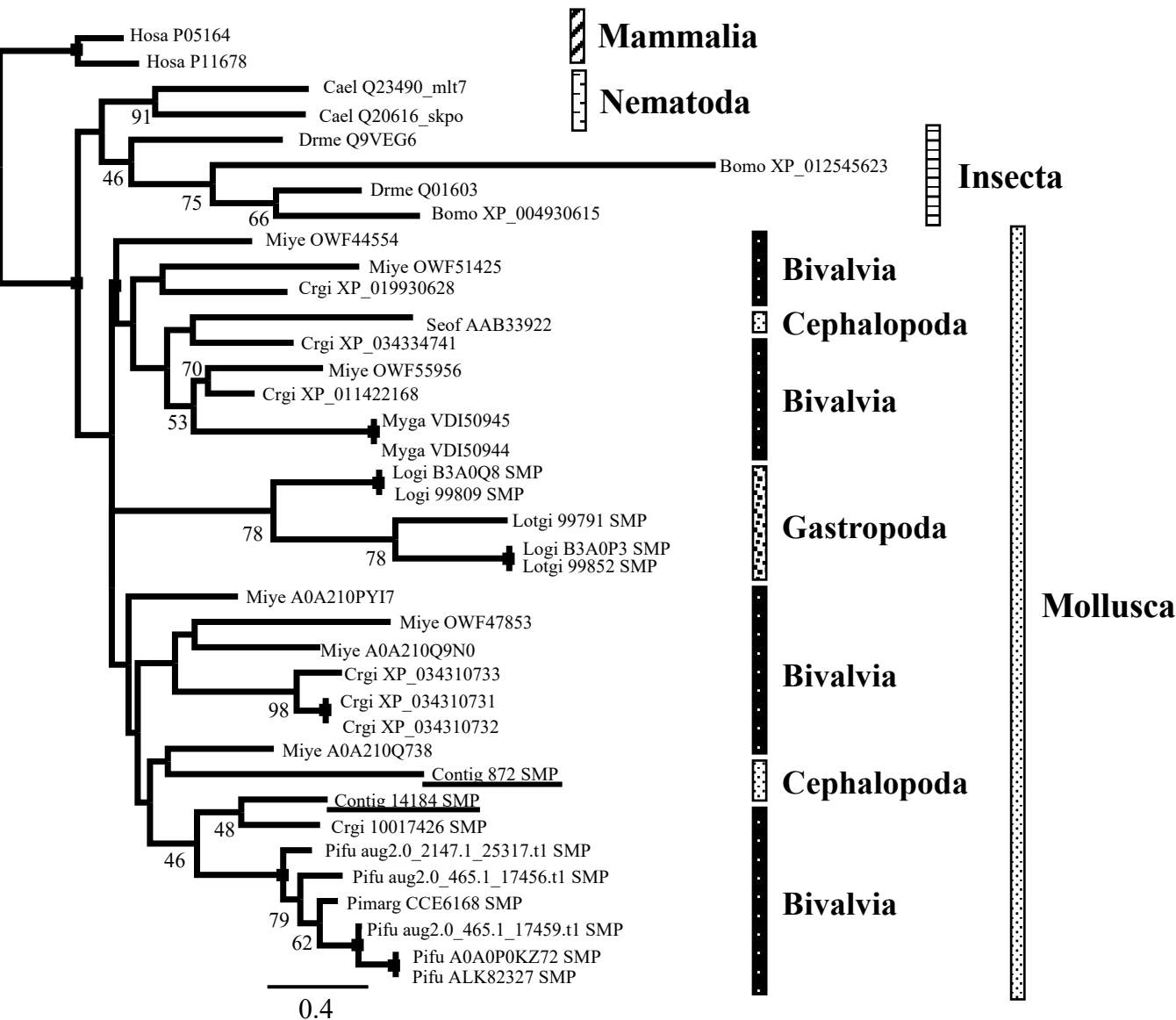

D

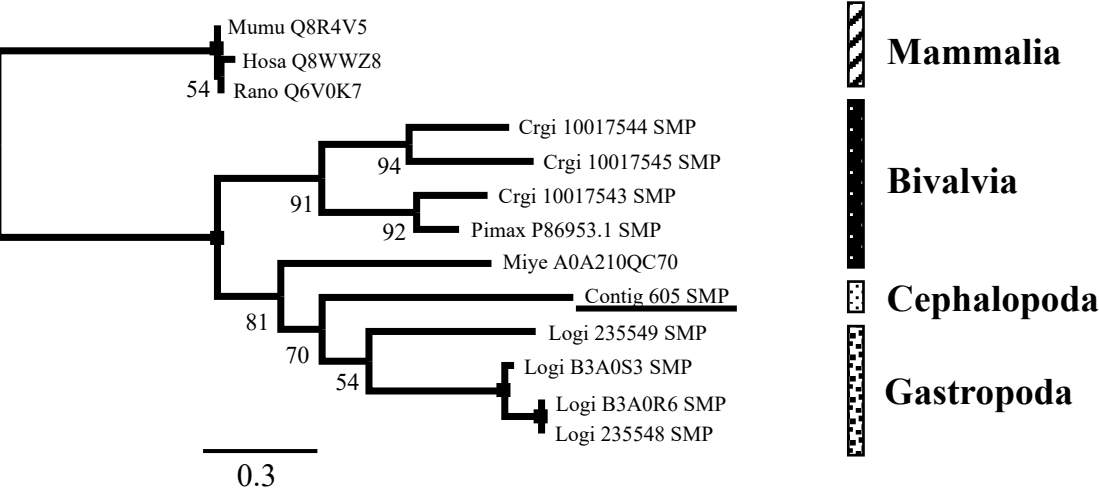

Supplement: Supplementary file 1 [file genes-12-01925-s001.zip › Supp_PDFs/4_Npo_Supp_FigS2.pdf]

Supplementary Fig. 1

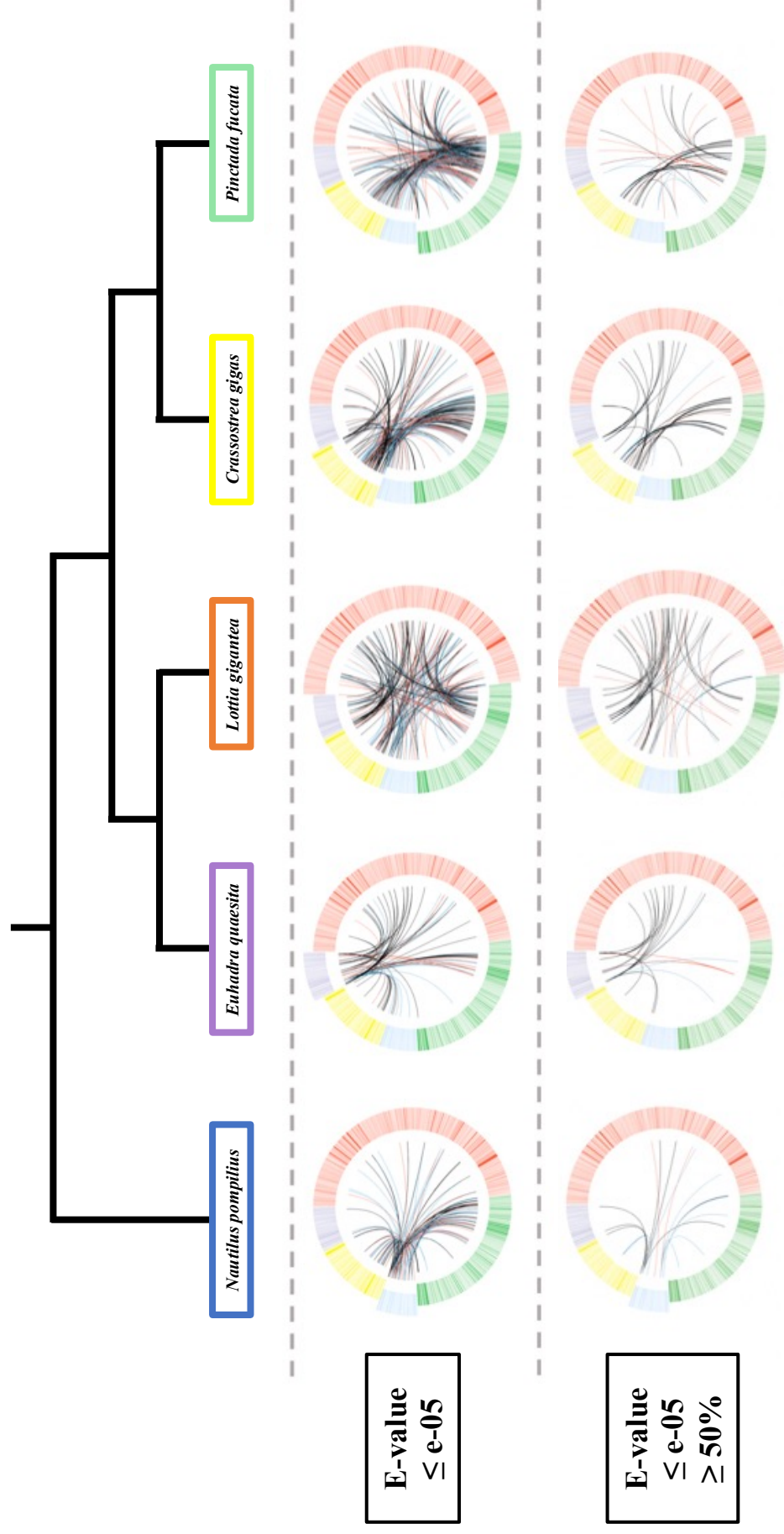

Supplementary Fig. 2

A

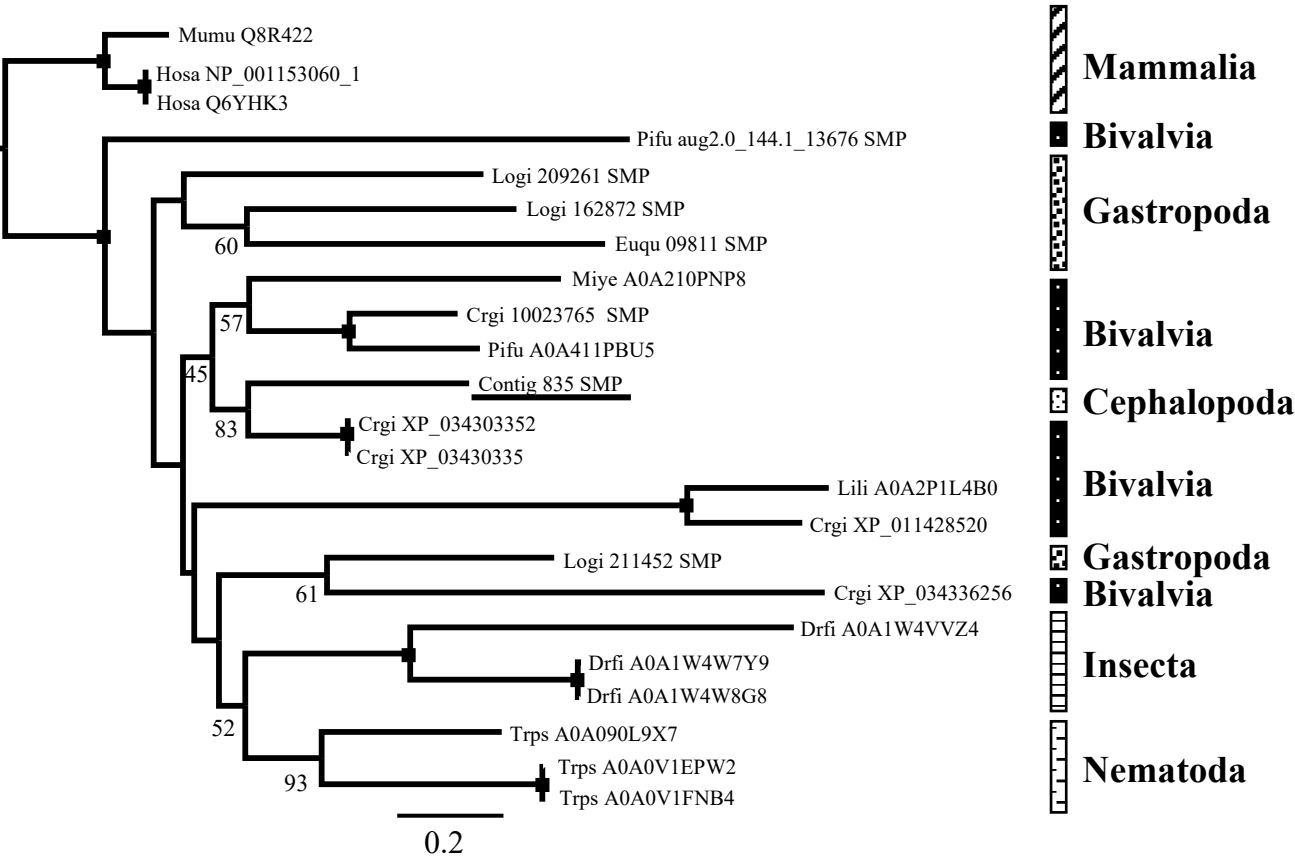

Supplementary Fig. 2

B

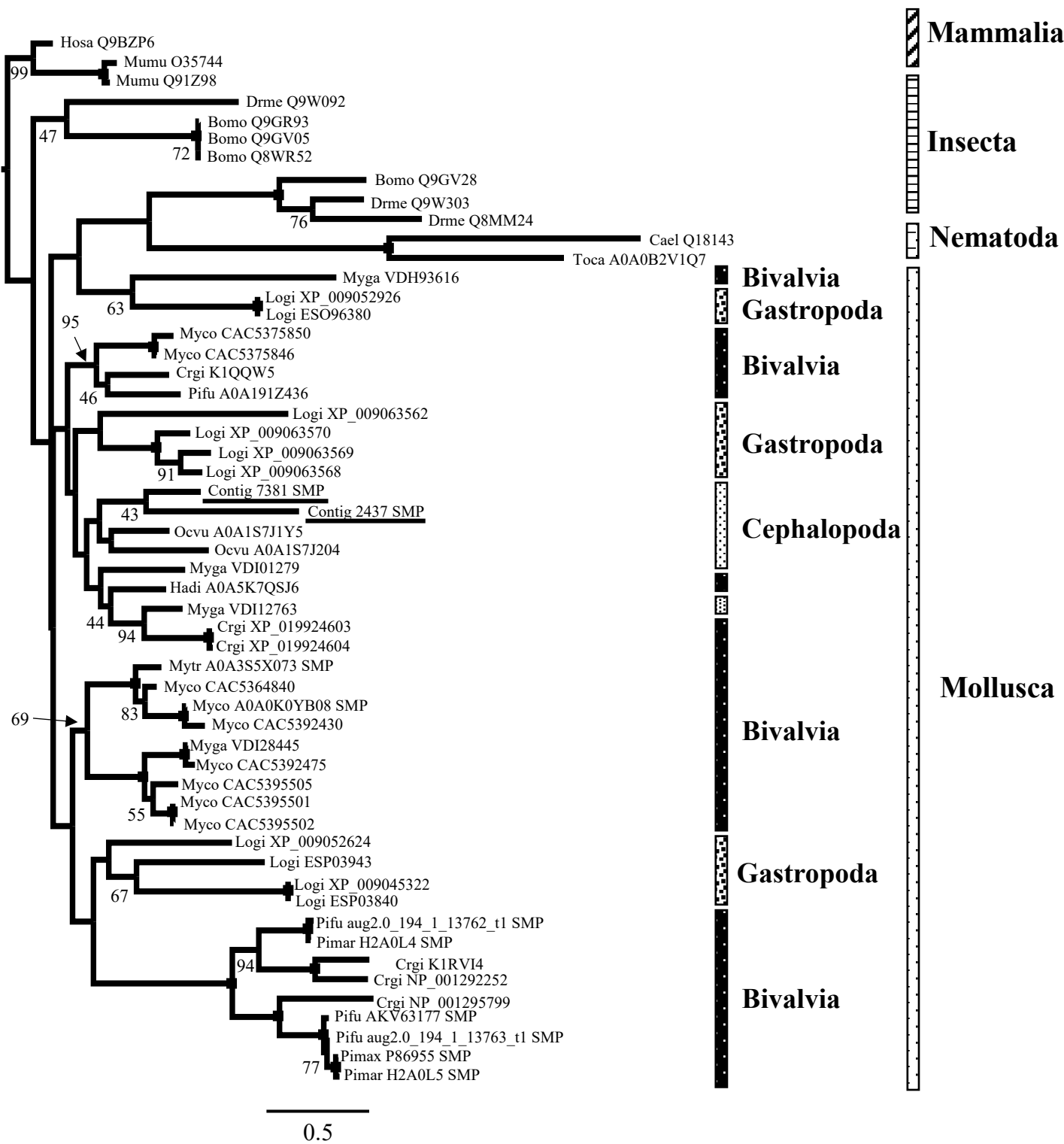

Supplementary Fig. 2

C

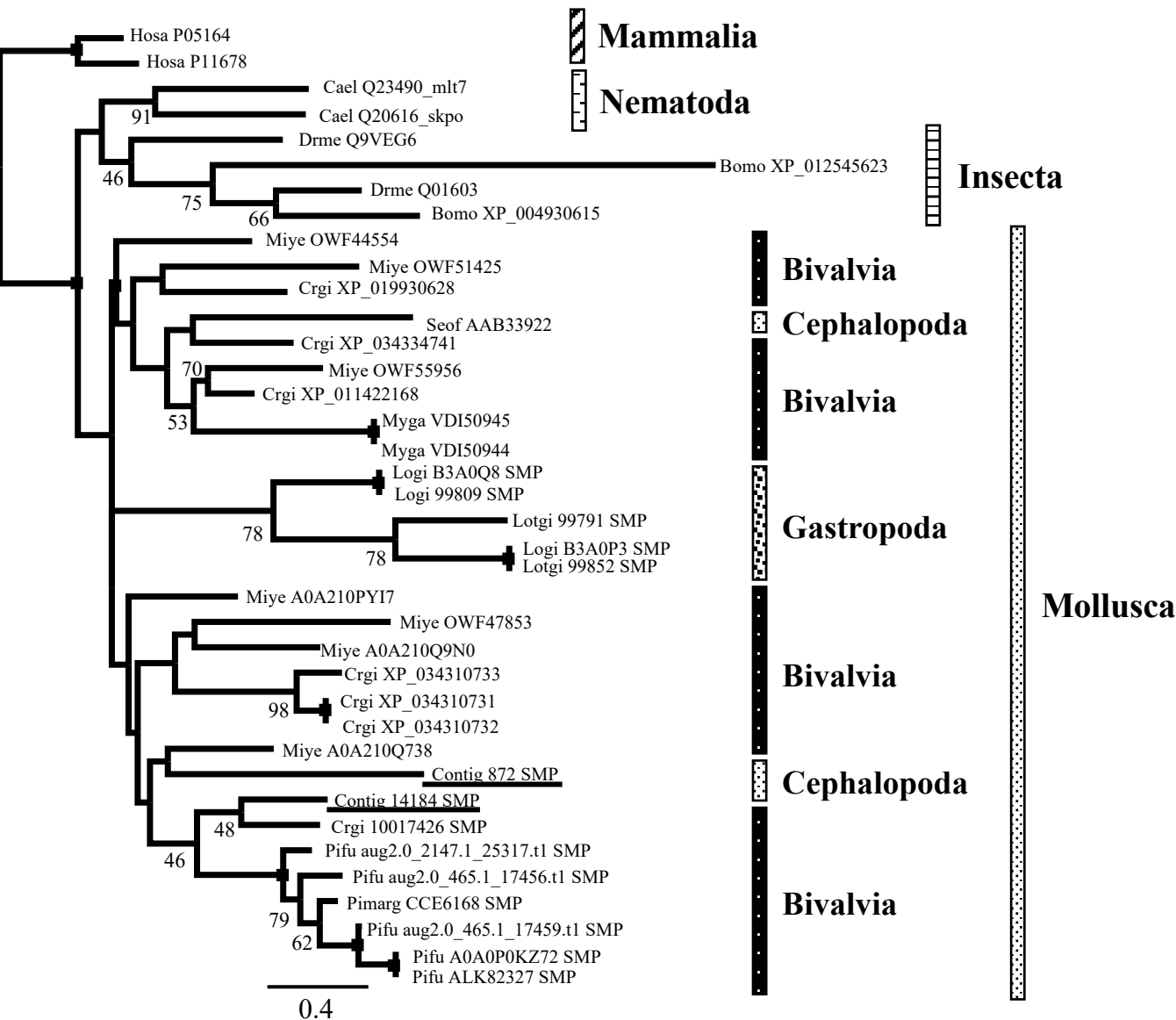

D

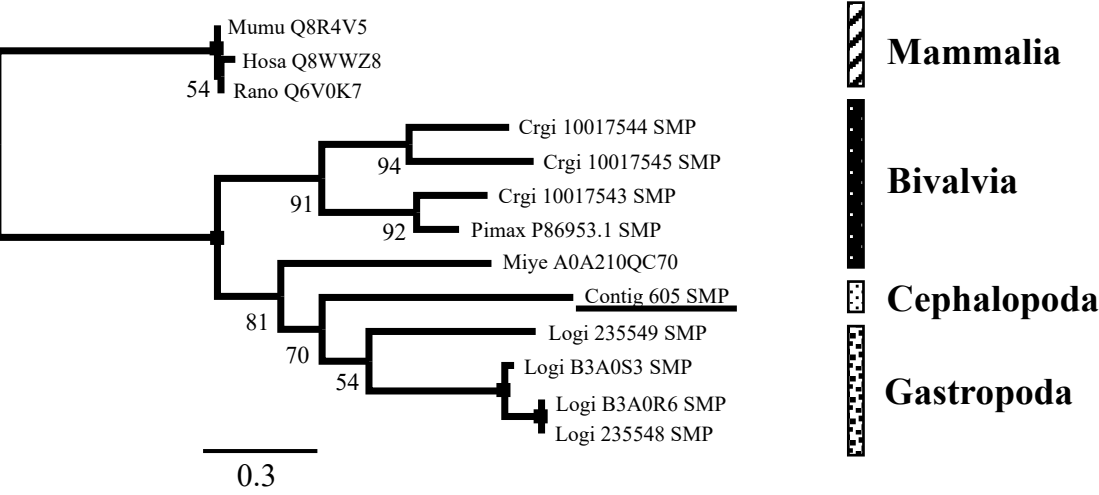

Supplement: Supplementary file 1 [file genes-12-01925-s001.zip › Supp_PDFs/4_Npo_Supp_FigS1.pdf]
